# Modulating BAP1 expression affects ROS homeostasis, cell motility and mitochondrial function

**DOI:** 10.18632/oncotarget.19872

**Published:** 2017-08-03

**Authors:** Lucie Hebert, Dorine Bellanger, Chloé Guillas, Antoine Campagne, Florent Dingli, Damarys Loew, Alice Fievet, Virginie Jacquemin, Tatiana Popova, Didier Jean, Fatima Mechta-Grigoriou, Raphaël Margueron, Marc-Henri Stern

**Affiliations:** ^1^ Department of Genetics and Biology of Cancers, INSERM U830, Institut Curie, PSL Research University, Paris 75248, France; ^2^ Department of Developmental Biology and Genetics, CNRS UMR 3215/INSERM U934, Institut Curie, PSL Research University, Paris 75248, France; ^3^ Mass Spectrometry and Proteomics facility, Institut Curie, PSL Research University, Paris 75248, France; ^4^ Department of Genetics, Institut Curie, Paris 75248, France; ^5^ INSERM UMR-1162, Paris 75010, France

**Keywords:** BAP1, tumor suppressor, de-ubiquitination, proteomics, protein stability

## Abstract

The tumor suppressor BAP1 associates with ASXL1/2 to form the core Polycomb complex PR-DUB, which catalyzes the removal of mono-ubiquitin from several substrates including histone H2A. This complex also mediates the poly-deubiquitination of HCFC1, OGT and PCG1-α, preventing them from proteasomal degradation. Surprisingly, considering its role in a Polycomb complex, no transcriptional signature was consistently found among *BAP1*-inactivated tumor types. It was hypothesized that BAP1 tumor suppressor activity could reside, at least in part, in stabilizing proteins through its poly-deubiquitinase activity. Quantitative mass spectrometry and gene expression arrays were used to investigate the consequences of *BAP1* expression modulation in the NCI-H226 mesothelioma cell line. Analysis of differentially expressed proteins revealed enrichment in cytoskeleton organization, mitochondrial activity and ROS management, while gene expression analysis revealed enrichment in the epithelial-to-mesenchymal transition pathway. Functional assessments in BAP1 inactivated, BAP1 wild-type and BAP1 catalytically dead-expressing NCI-H226 and QR mesothelioma cell lines confirmed alteration of these pathways and demonstrated that BAP1 deubiquitinase activity was mandatory to maintain these phenotypes. Interestingly, monitoring intracellular ROS levels partly restored the morphology and the mitochondrial activity. Finally, the study suggests new tumorigenic and cellular functions of BAP1 and shows for the first time the interest of studying the proteome as readout of BAP1 inactivation.

## INTRODUCTION

The deubiquitinating enzyme (DUB) BRCA1-associated protein 1 (BAP1) is a tumor suppressor inactivated in a variety of cancers, including cutaneous and uveal melanoma, pleural mesothelioma, and renal cell carcinoma [1–5]. *BAP1* germline deleterious mutations are responsible for a cancer predisposition syndrome prone to the aforementioned tumor types and probably others [6–8].

BAP1 is a deubiquitinase belonging to the ubiquitin carboxyl hydrolase (UCH) family and its enzymatic activity was first shown to target histone H2A. Its ortholog Calypso is classified as a Polycomb protein essential for maintaining *Hox* gene repression during *Drosophila* embryo development. Calypso interacts with Asx (Additional sex comb) to form the Polycomb Repressive DeUBiquitinase complex (PR-DUB) [9]. This complex is conserved in mammals, although *Hox* gene expression was not reported to be altered in *Bap1-*inactivated mice [10]. Moreover, BAP1 was reported to interact with several transcription-related proteins including YY1 (Ying-Yang 1), HCFC1 (Host Cell Factor-1), FOXK1/2 (Forkhead box 1 and 2), KDM1B (lysine (K)-specific demethylase 1B) and MBD5 and MBD6 (methyl-CpG binding domain protein 5 and 6, respectively) [11–15]. From the aforementioned results, *BAP1* inactivation is expected to affect transcription regulation, either through direct gene expression dysregulation or chromatin structure perturbation. Consistently, *BAP1* loss was shown to lead to EZH2-mediated transformation [16], although this observation might be context specific [17]. Surprisingly, comparative transcriptomic analyses of renal carcinoma and mesothelioma failed to identify a consistent gene expression signature for *BAP1*-inactivated tumors [3, 15, 18].

BAP1 is able to poly-deubiquitinate lysine 48-linked ubiquitin chains of HCFC1 [13], OGT (O-GlcNAc transferase) and PGC-1α (Peroxisome proliferator-activated receptor-gamma coactivator) [19] in mammals, protecting them from proteasomal degradation [10, 14, 19].

Both the lack of consistent transcriptomic consequences and the identification of several poly-deubiquitinated targets led to the hypothesis that BAP1 tumor suppressor function could reside, at least in part, in its ability to stabilize target proteins. To test this hypothesis, we investigated the consequences of *BAP1* expression modulation at transcriptome and proteome levels. A signature of cell morphology, migration and invasion was found by both approaches. On the contrary, only protein enrichment analysis revealed an impact on mitochondrial respiratory function. Functional assessment in two mesothelioma cell line models confirmed alterations of cell morphology, migration and invasion as well as alteration of respiratory function. We propose that the increase of intra-cellular levels of reactive oxygen species (ROS) upon wild-type re-expression of a catalytically active BAP1 is at least in part responsible for morphologic changes, acquired invasive capacities, and respiratory defects.

## RESULTS

### Transcriptome and proteome analyses identified two major biological pathways associated with modulation of *BAP1* expression

**Figure 1:**
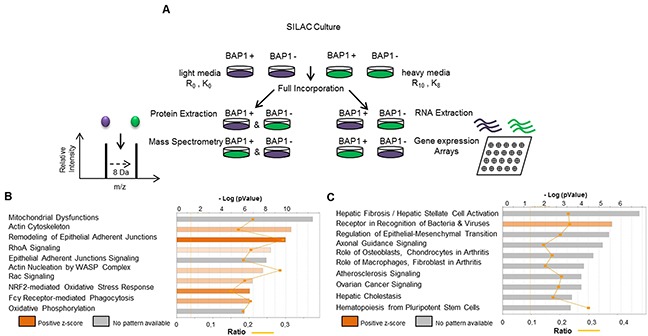
SILAC-based proteomics approach (SILAC/MS) reveals two major signatures associated with *BAP1* expression **(A)** Description of the experiment procedure. NCI-H226 cell line expressing either an empty vector (EV) or wild-type BAP1 (BAP1^wt^) were cultured simultaneously in “light” (purple) and “heavy” (green) SILAC media. Gene expression arrays (Affymetrix®) and tandem mass spectrometry (MS/MS) were then performed in parallel in SILAC-treated cells material. After confirmation of >98% marked amino-acid incorporation by mass spectrometry analysis, equal amount of proteins were mixed and trypsin-digested. Peptides were quantified and identified by nano-LC-MS/MS. **(B** and **C)** Ingenuity Pathway Analysis (IPA) software was used to examine the biological processes enriched by differentially expressed proteins (B) and differentially expressed genes (C) after BAP1 re-expression.

In order to analyze the effects of *BAP1* expression modulation on the proteome, SILAC/MS (Stable Isotope Labelling Amino acid in Cell culture coupled with tandem Mass Spectrometry) and gene expression arrays were performed on NCI-H226 cell line, which is deleted for *BAP1*, and is a classic model to assess BAP1 localization and catalytic activity [20, 21]. It was not clear from literature whether NCI-H226 was derived from a mesothelioma or a lung carcinoma (ATCC, CRL-5826) [20, 22]. We confirmed its mesothelioma origin by positioning it within the mesothelioma cluster of the Cancer Cell Line Encyclopedia, far from squamous and from non-small cell lung adenocarcinoma (Supplementary Figure 1). NCI-H226 was stably complemented with pCDH1 lentiviral vector alone (EV for Empty Vector) (pCDH1_EV) or expressing wild-type *BAP1* (pCDH1_BAP1^wt^). Both pCDH1_BAP1^wt^ and pCDH1_EV cell lines were grown in heavy and light isotope media in order to obtain reciprocal experiments (Figure 1A). To evaluate the impact of *BAP1* expression modulation on the proteome, protein quantification was evaluated by SILAC/MS. Differential protein accumulation was defined by (i) a consistent unbalanced ratio between NCI-H266 cell lines expressing BAP1^wt^ and not expressing BAP1 in the two reciprocal experiments; (ii) an adjusted p-value less than 0.05 to assess ratio significance; (iii) and a mean value ratio greater than 1.2 or less than 0.8 (Supplementary Table 1). On this basis, 1098 proteins displayed differential accumulation, including 556 over-represented and 542 under-represented proteins in the presence of pCDH1_BAP1^wt^. Ingenuity Pathway Analysis (IPA) was applied for these proteins and revealed significant enrichments of two main pathways: cell morphology and motility, and mitochondrial functions. Cell morphology and motility was characterized by actin cytoskeleton and nucleation signaling, epithelial adherent junction signaling and RhoA and Rac activation signaling canonical pathways. Mitochondrial functions were characterized by oxidative phosphorylation, NRF2-mediated oxidative stress response and mitochondrial dysfunction canonical pathways (Figure 1B). Each canonical pathway was represented by a high number of differentially expressed proteins, with a total of 118 proteins involved in the two major pathways (Supplementary Table 2 and Supplementary Figure 2). For instance, α-actinin 1 and 4, responsible for actin filament crosslinking, actin-related or actin-binding proteins including the ARP and ACTR families, as well as caveolin, were found 1.5 to 2.5-fold increased when the NCI-H226 cell line expressed pCDH1_BAP1^wt^. On the contrary, N-Cadherin (CDH2) was 2.6-fold decreased in pCDH1_BAP1^wt^ expressing cells (Supplementary Figure 2A). The mitochondrial functions were represented by at least 20 proteins involved in the five complexes of the mitochondrial respiratory chain (CI, CII, CIII, Cyt c and CV), which had a 1.3 to 2.1-fold reduced expression when pCDH1_BAP1^wt^ was expressed. Furthermore, several DnaJ homolog family members, which interact with chaperone proteins and participate in mitochondrial integrity, had a similar reduced expression in pCDH1_BAP1^wt^ expressing cells (Supplementary Figure 2B). Finally, a high number of proteins involved in detoxification, anti-oxidation, and / or being NRF2 targets were found significantly over-expressed by SILAC/MS in a pCDH1_BAP1^wt^ expression context, suggesting an increased intra-cellular ROS level (Supplementary Figure 2C).

Differential gene expression analysis identified 734 significantly differentially expressed genes according to these criteria: (i) correct annotation of probes and corresponding genes, (ii) absolute value of log_2_ of fold-change |log_2_ (FC)|>1, (iii) standard deviation (SD) between the replicates: SD <0.3 and (iv) same direction of dysregulation between two replicates (Supplementary Table 3). IPA of these differentially expressed genes revealed significant enrichments for the epithelial-mesenchymal transition pathway (Figure 1C), suggesting that motility and cell morphology are altered both at the transcriptomic and at the proteomic level. The others top canonical pathways were related to fibrosis, arthritis and atherosclerosis, and highly represented by extra-cellular gene coding proteins and matrix-metalloproteinase, which could also reflect the cell morphology and migratory changes. To evaluate the involvement of transcriptomic deregulation in the pathways observed at the protein level, we compared the differentially expressed genes and their corresponding protein level. Only 55 out the 1098 (5%) differentially enriched proteins were also differentially expressed at the mRNA level, suggesting that the pathways highlighted by the proteomic analysis were mainly due to post-transcriptomic dysregulations (Supplementary Table 4). Of note, these 55 genes encode proteins mainly involved in either mitochondrial integrity or cellular morphology.

### BAP1 deubiquitinase activity is associated with cytoskeleton reorganization and increased invasive/migratory capacities

In order to confirm the phenotypes predicted by IPA on the SILAC-based proteomics data and to determine the involvement of BAP1 catalytic activity, NCI-H226 and QR (also known as MPM_33 [23]) mesothelioma cell lines were complemented with pBABE retroviral vector alone (EV), pBABE expressing a wild-type BAP1 protein (BAP1^wt^), or the same vector expressing a catalytically dead BAP1 protein carrying the C91S point mutation (BAP1^C91S^) (Supplementary Figure 3A&3B).This mutation specifically affects the catalytic cysteine and prevents BAP1 deubiquitinase activity [11].

**Figure 2:**
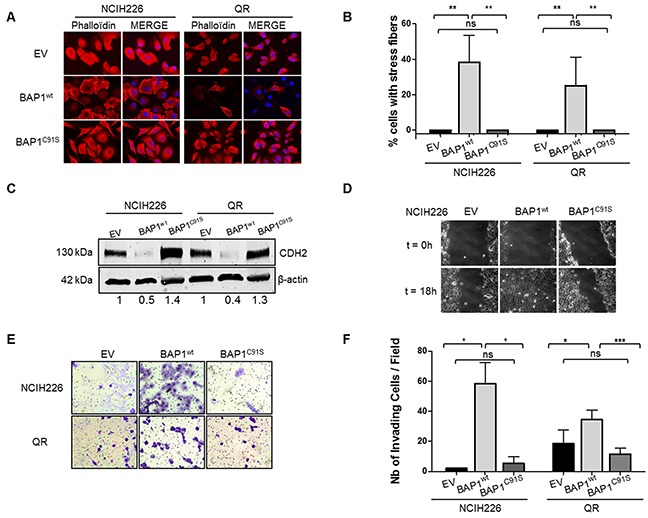
BAP1 deubiquitinase activity is associated with morphological changes and acquired invasive/migratory capacities **(A)** Staining with DICT-phalloïdin (red) to observe F-actin fibers. Nuclei were counterstained with DAPI. Magnification x400. **(B)** Box plot representing the percentage of cells with F-actin fibers. **(C)** Western blot quantifying CDH2. β-actin is used as loading control. **(D)** Representative photographs of wound-healing assays at 0 and 18h after creating a wound (magnification x100). **(E)** Representative photographs of invasion in Boyden chambers after 24h (magnification x200). **(F)** Bar graphs based on quantitative data from E. Data are mean ± SEM. *: P < 0.05, **: P < 0.01, ***: P < 0.001 versus Empty Vector (EV). Each experiment was repeated at least three times. EV, BAP1^wt^, BAP1^C91S^: NCI-H226 and QR transfected with empty, wild-type BAP1 and deubiquitinase dead BAP1 vectors, respectively.

We examined the global organization of the cytoskeleton of the isogenic cell lines. Phalloïdin staining revealed prominent cortical actin and actin stress fibers in more than 40% and 25% of NCI-H226 and QR cell lines expressing BAP1^wt^, respectively. Only a diffuse signal with no stress fibers was observed in cell lines expressing either EV or BAP1^C91S^ (Figure 2A&2B). Western blot analysis confirmed a 2-fold decrease N-cadherin (CDH2) expression in NCI-H226 and QR cells expressing BAP1^wt^ and a slight increase was observed in cells expressing catalytically dead BAP1 (Figure 2C). Immunofluorescence revealed an expected CDH2 membrane localization in control EV cells and upon expression of BAP1^C91S^. By contrast, its localization was mainly cytoplasmic in wild type BAP1 expressing cell lines (Supplementary Figure 4).

Cell migration capacities were then evaluated by wound healing assay. Motility was more than 2-fold increased in NCI-H226 BAP1^wt^-expressing cell line compared to the two other genotypes. Indeed, the scratch was completely filled after an average of 15 hours for BAP1^wt^ cell lines (Figure 2D), whereas it was largely incomplete in both EV and BAP1^C91S^ expressing cell lines after 30 hours. This indicates a dramatic increase of cell migratory capacities when a functional BAP1 protein is expressed. Boyden chambers coated with Matrigel® were used to investigate invasive capacities. Cells expressing BAP1^wt^ had a 10-fold and a 2-fold increase of invading cells as compared either to EV or BAP1^C91S^ in NCI-H226 and QR, respectively (p<0.05, Figure 2E&2F). Of note, proliferation curves of the NCI-H226 isogenic cell lines showed comparable proliferation capacities in the time range of the assays, although a significant growing advantage of BAP1^wt^ over EV or BAP1^C91S^ is detected from 4 to 7 days post-seeding (Supplementary Figure 3C).

### BAP1 deubiquitinase activity is associated with reduced respiratory capacities

**Figure 3:**
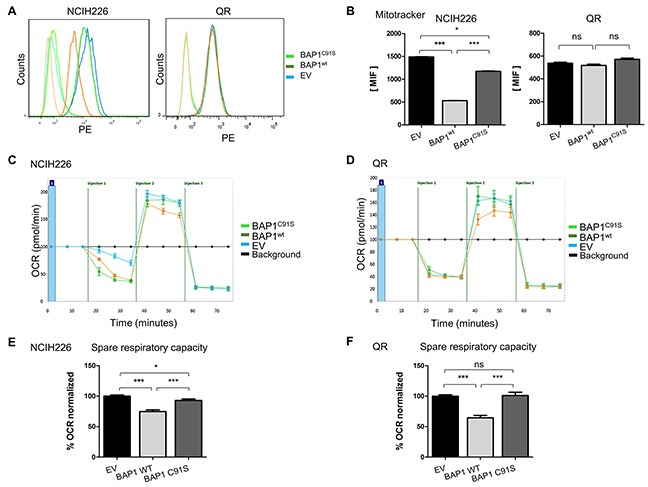
BAP1 deubiquitinase activity is associated with decrease of mitochondrial active mass and reduced respiratory capacities **(A)** Mitochondrial membrane potential was measured by FACS analysis using MitoTracker Red, reflecting mitochondrial activity and respective associated DMSO controls. **(B)** Bar graphs represent a quantification of mean intensity fluorescence (MIF). **(C** and **D)** The oxygen consumption rate (OCR) of cells is measured by the Seahorse XF-96 extracellular flux analyzer for NCI-H226 and QR (C and D, respectively) after the addition of three inhibitors: oligomycin (1μM or 1.5μM for NCI-H226 and QR cell lines respectively), FCCP (Carbonyl cyanide-4 (trifluoromethoxy) phenylhydrazone) (0.5 μM or 1μM for NCI-H226 and QR cell lines respectively), and a mix of rotenone + antimycin A (1 μM each), which are serially injected to measure ATP production, maximal respiration, and non-mitochondrial respiration, respectively.(i.e. injections). **(E** and **F)** Bar graphs are quantifications of respiratory capacity measurements for NCI-H226 and QR (C and D, respectively) (i.e. maximal respiration minus basal respiration). Data are mean ± SEM. *: P < 0.05, **: P < 0.01, ***: P < 0.001 versus Empty Vector (EV). Each experiment was repeated at least three times. EV, BAP1^wt^, BAP1C91S: NCI-H226 and QR transfected with empty, wild-type BAP1 and deubiquitinase dead BAP1 vectors, respectively.

Mitochondrial dysfunction was one of the main pathways highlighted after BAP1 re-expression, and SILAC/MS revealed a high number of proteins involved in the 5 respiratory complexes with a 2-fold reduction in a BAP1^wt^ expression context (Figure 1B, Supplementary Figure 2B). Of note, the change of ATP5A1, UQRC2, and SHBD protein expression could not be confirmed when using the Total OXPHOS Antibody Cocktail (data not shown). To measure active mitochondrial network content, we used the MitoTracker Red probe that accumulates in active mitochondria by recognition of depolarization in the inner mitochondria membrane. Flow cytometry analysis revealed that NCI-H226 expressing BAP1^wt^ has a significantly decreased number of active mitochondria (3 and 2.6 fold decreased BAP1^wt^ vs EV and BAP1^C91S^, respectively; p<0.001. Of note, the expression of BAP1^C91S^ resulted in a decrease of active mitochondria compared to the empty vector, p<0.05). On the contrary, QR cell line shows no significant difference of active mitochondria content, suggesting that the phenotype observed in NCI-H226 might be cell line specific (Figure 3A&3B). Furthermore, membrane depolarization difference could not be confirmed when using different probes such as TMRM (data not shown) supporting the fact that mitochondrial function alteration is not due to membrane depolarization. To evaluate the respiratory capacities, the Seahorse® technique was used on both NCI-H226 and QR isogenic cell lines. Cells were seeded with adjustment of cell number in order to reach around 80% of confluence and obtain a similar basal oxygen consumption rate (OCR) (Figure 3C&3D). The mitochondrial specific respiratory capacities calculation (Maximal OCR minus Basal OCR) revealed a 1.3 and 1.5-fold decrease of respiratory capacities for NCI-H226 and QR expressing BAP1^wt^ as compared to either EV or BAP1^C91S^, respectively (p<0.001, Figure 3E&3F). Of note, the expression of BAP1^C91S^ resulted in a decrease of respiratory capacities in NCI-H226 cell line compared to the empty vector, Figure 3E, p<0.05), while EV and BAP1^C91S^ have similar respiratory capacities in QR cell line (Figure 3F). Extracellular acidification rate (ECAR), reflecting cell glycolysis, was measured and compared between NCI-H226 cells expressing empty vector and BAP1^wt^ (Supplementary Figure 5A), but no significant difference was observed for neither their glycolytic capacity nor their glycolytic reserve (Supplementary Figure 5B&C).

### BAP1 deubiquitinase activity is associated with increased intra-cellular ROS

**Figure 4:**
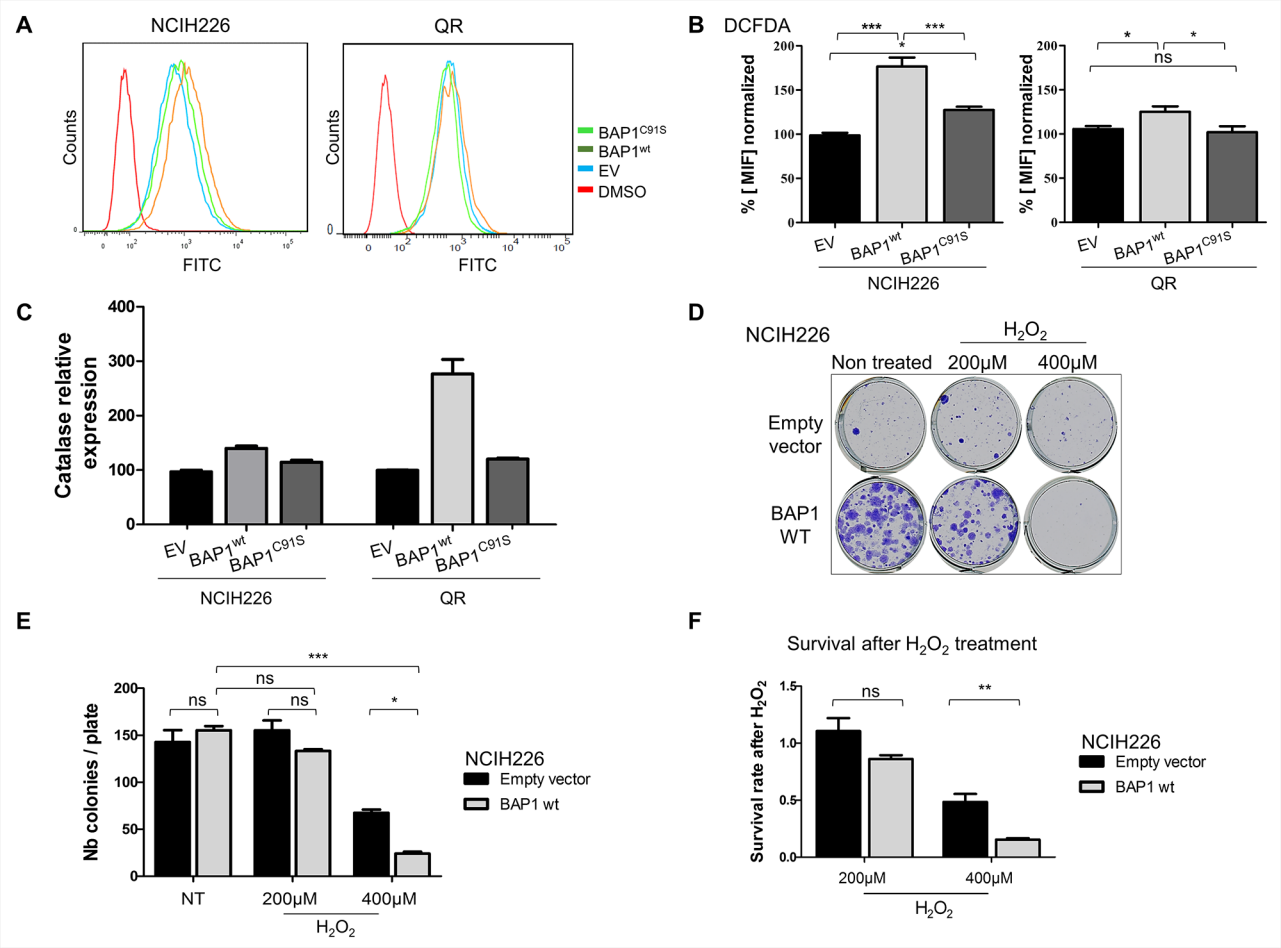
BAP1 deubiquitinase activity is associated with increased intra-cellular ROS level and sensitivity to oxidative stress **(A)** Intracellular ROS level evaluation by measurement of DCFHDA (10 μM) probe fluorescence by flow cytometry, and DMSO negative control. **(B)** Bar graphs showing the data based on A. **(C)** *Catalase* expression measured by qPCR, normalized by *TBP* gene expression level. **(D)** Colony Formation Assay after H_2_O_2_ treatment. Cells were seeded and treated the day after for 8 hours with 200μM or 400μM of H_2_O_2_. **(E)** Colony counting after crystal violet fixation/coloration. **(F)** Survival after H_2_O_2_ treatment was calculated by doing the ratio between the number of colony of H_2_O_2_ treated cells and the number of colony of non-treated cells. Ratios were normalized with the number of colonies of non-treated NCI-H226 expressing empty vector. Data are mean ± SEM. *: P < 0.05, **: P < 0.01, ***: P < 0.001 versus control (EV). Each experiment was repeated at least three times. EV, BAP1^wt^, BAP1^C91S^: NCI-H226 and QR transfected with empty, wild-type BAP1 and deubiquitinase dead BAP1 vectors, respectively.

Accumulation of numerous proteins involved in oxidative stress response was modulated by BAP1 expression (Supplementary Figure 2C). To further investigate this pathway alteration, intra-cellular ROS level was quantified by flow cytometry using carboxy-H_2_DCFDA. Fluorescent DCFDA reflected a 1.8-fold and 1.3-fold increase of intracellular ROS level in BAP1^wt^ cells compared to EV cells for NCI-H226 and QR, respectively (p<0.001 and p<0.05, Figure 4A&4B) and a 1.5-fold and 1.4-fold increased ROS level compared to BAP1^C91S^ cells for NCI-H226 and QR, respectively (p<0.001 and p<0.05, Figure 4A&4B). Of note, the expression of BAP1^C91S^ resulted in a significant increase of ROS level in NCI-H226 cell line compared to the empty vector (p<0.05; Figure 4B) while EV and BAP1^C91S^ have similar ROS levels in QR cell line (Figure 4A&4B). Catalase is directly involved in ROS management and was found differentially expressed in SILAC/MS (ratio = 3.4, Supplementary Table 1) and gene expression array (log_2_ (FC) = 1.3, Supplementary Table 3). *CAT* expression measured by qPCR confirmed a 1.5-fold and 3-fold increased expression level associated with BAP1^wt^ expression compared to either EV or mutant BAP1^C91S^ expressing cells in NCI-H226 and QR, respectively (Figure 4C). Surprisingly, no other NRF2 target gene expression was increased in this high ROS cellular context (Supplementary Figure 6). NCI-H226 expressing EV or mutant BAP1^C91S^ showed significantly lower intracellular ROS levels compared to BAP1^wt^ (p<0.05; Figure 4B), and ROS levels did not fluctuate under antioxidant treatment (Supplementary Figure 7), and EV and mutant BAP1^C91S^ expressing NCI-H226 show similar level of ROS management proteins. Thus, further experiments were performed comparing NCI-H226 expressing EV and BAP1^wt^. The cellular sensitivity to exogenous oxidative stress is strongly dependent on the endogenous redox status [24]. Therefore, NCI-H226 control or expressing BAP1^wt^ were exposed to high extra-cellular oxidative stress, and survival was measured as readout of intracellular redox management. Cells were treated with either 200μM or 400μM H_2_O_2_ for 8 hours and ROS sensitivity was evaluated by Colony Formation Assay (CFA). Fourteen days after seeding, colonies were counted. Both cells expressing EV and BAP1^wt^ had similar colony formation capacities (146 and 152 colonies, respectively; Figure 4D&4E). However, a difference in colony size was observed, where NCI-H226 cells expressing BAP1^wt^ had larger colonies, which is consistent with the cell proliferation capacities shown in Supplementary Figure 3C. After 200uM H_2_O_2_ treatment, the change in colony number was not significant for both EV and BAP1^wt^ expressing cells compared to their non-treated counterpart (Figure 4D&E). On the contrary, 400μM H_2_O_2_ treatment lead to dramatic decrease of colony number. Importantly, BAP1^wt^ expressing cells were significantly more affected by H_2_O_2_ treatment than control EV cells (p<0.05, Figure 4E). In order to quantify cell sensitivity to an increased oxidative stress, survival after H_2_O_2_ treatment was calculated. Number of colonies after treatment was normalized to the basal colony capacity (number of colony after treatment divided by number of colony in non-treated cells). After 400μM H_2_O_2_, BAP1^wt^ cells had a 50% survival rate and EV cells had 15% survival rate, which is a 3-fold difference (p<0.01, Figure 4F). These results corroborate DCFDA measurements and strongly argue that re-expression of BAP1^wt^ in mesothelioma cell lines increases intracellular ROS level, leading to a higher sensitivity to additional exogenous oxidative stress.

### BAP1-related increased intracellular ROS level is involved in both morphologic and respiratory capacities alterations

**Figure 5:**
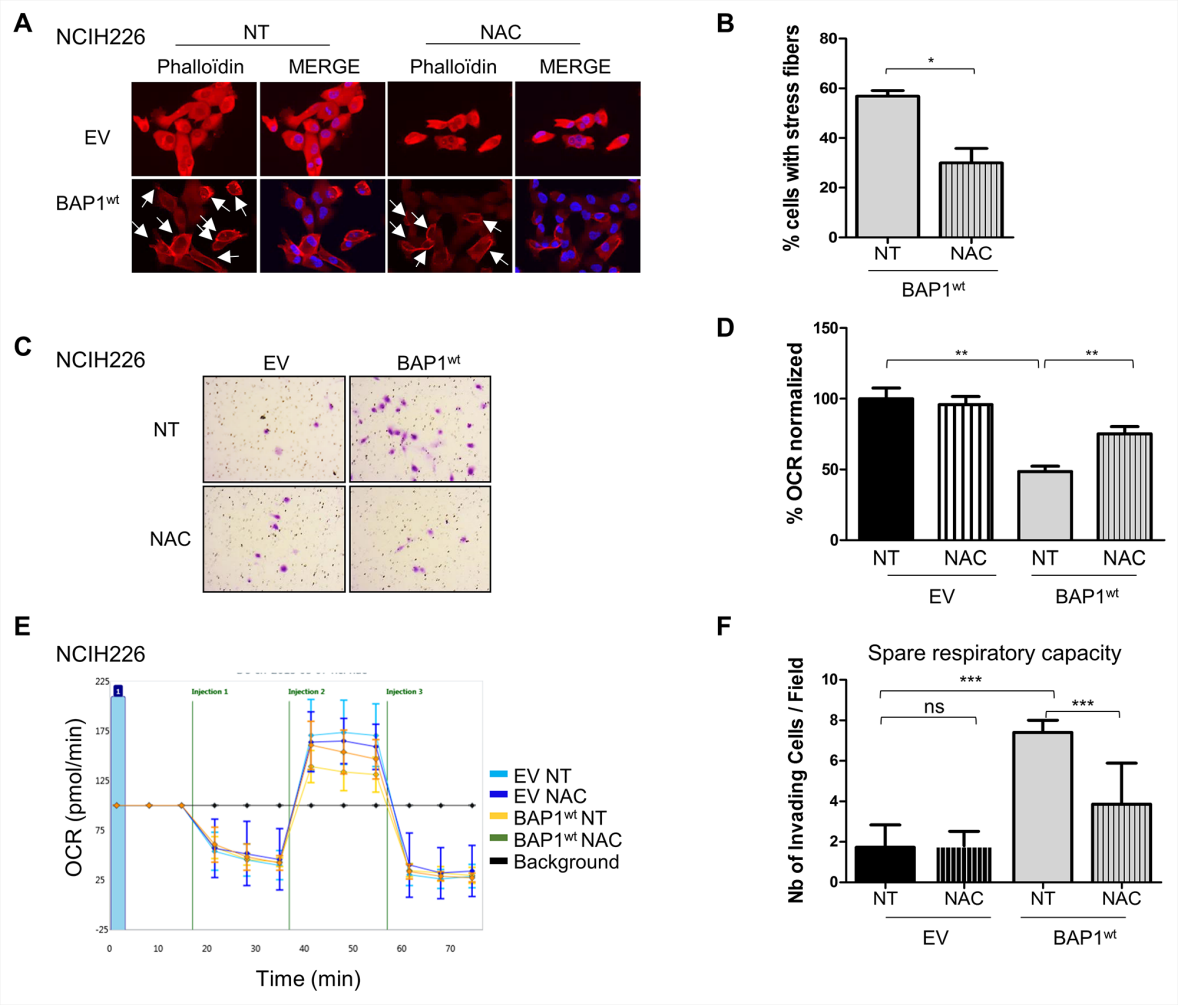
BAP1-related increased ROS level is involved in both morphological and metabolic changes in NCI-H226 cell line **(A)** Staining with DICT-phalloïdin (red) to observe F-actin fibers in cells treated with 500nM NAC (N-acetyl-cysteine) during 10 days versus non-treated cells (NT). Nuclei were counterstained with DAPI. Magnification x400. Cells presenting with stress fibers are indicated by white arrows. **(B)** Bar graph representing the percentage of cells with F-actin fibers counted in 7 images in two independent experiments. **(C)** Representative images of invasion in Boyden chambers taken 24h after seeding on cells treated with 500nM NAC during 10 days versus NT. **(D)** Bar graphs based on quantitative data from C. **(E)** The oxygen consumption rate (OCR) of cells measured by the Seahorse XF-96 extracellular flux analyzer after addition of 3 inhibitors at different time points (i.e. injections) on cells treated with 500nM NAC for 10 days versus non-treated cells (NT). **(F)** Bar graphs are quantifications of respiratory capacity measurements (i.e. maximal respiration minus basal respiration). Data are mean ± SEM. *P < 0.05 versus non treated empty vector (EV). Each experiment was repeated at least three times. EV, BAP1^wt^: NCI-H226 transfected with empty and wild-type BAP1 vectors, respectively.

In a normal cellular context, increase of intracellular ROS level should result in mitosis, apoptosis and necrosis, but in a tumor context, this phenomenon has been shown to be involved in acquired tumorigenic properties such as invasiveness [25] and mitochondrial respiration defects [26]. To evaluate the effect of BAP1-related ROS increase in NCI-H226 mesothelioma model, isogenic cell lines were treated with low quantity of the anti-oxidant molecule N-acetylcysteine (NAC) for 10 to 15 days. The antioxidant effect of NAC was monitored by *CAT* expression, and showed a 1.5-fold decrease in NAC-treated BAP1^wt^ cell line (Supplementary Figure 7). Cell morphology was then assessed by phalloïdin labelling and microscopy. Cell line expressing BAP1^wt^ had a 2-fold decrease of number of cells presenting with cortical actin and stress fibers, as indicated by the cells marked with an arrow, before and after NAC treatment (7/13 vs 6/24). The graph illustrates a compilation of 7 images for each condition, (p<0.05, Figure 5A&5B) and no effect was observed in EV cells. NAC treatment also revealed a 2-fold decrease of invasive capacities of cell expressing BAP1^wt^ (p<0.01), while confirming the difference between the two isogenic non-treated cell lines (4-fold; p<0.01, Figure 5C&5D). To evaluate the effect of increased ROS level on mitochondrial functions, OCR was measured after NAC treatment and revealed a significant 1.5-fold increase of respiratory capacities in BAP1^wt^ cells (p<0.01), while no change was observed for EV cells (1.6-fold; p<0.001, Figure 5E&5F). Of note, this experiment confirmed the aforementioned difference between EV and BAP1^wt^ non-treated cell lines.

## DISCUSSION

Tumor suppressor functions are difficult to decipher because of the complexity of gene interaction networks and their associated functions, and because of the influence of the tumor genetic background. Hence, no consistent transcriptional signature has been associated to *BAP1* inactivation among the *BAP1*-associated tumors [4, 18], although histone H2A deubiquitination and interactions with numerous transcriptomic factors are strongly supported by an abundant literature. In spite of this, considering the capacity of BAP1 to poly-deubiquitinate several protein targets, and that BAP1 has been shown to prevent these targets from proteasomal degradation, it was hypothesized that the BAP1 tumor suppressor trait could reside at least in part in the regulation of protein stability in the nucleus through its deubiquitinase function.

Parallel investigation of transcriptomic and proteomic consequences of BAP1 expression modulation revealed major protein dysregulations. Surprisingly, BAP1-mediated mRNA and protein regulation have a modest overlap and only a subset of the deregulated proteins after BAP1 expression modulation displays a corresponding change of mRNA accumulation. This result highlights the importance of studying the proteome as readout for BAP1 function. These alterations were confirmed *in vitro* using NCI-H226 and QR mesothelioma cell lines. Robust phenotypes were identified, and the use of catalytically dead BAP1 directly linked the phenotypes observed to BAP1 deubiquitinase activity. However, mutant BAP1 expression led to some phenotypic differences, suggesting that the catalytically inactive BAP1 may maintain some biological activities – possibly a scaffold or a dominant negative protein.

Our study mainly highlighted cytoplasmic con-sequences of BAP1 expression modulation, possibly due to an active cytoplasmic fraction of BAP1, Supplementary Figure 9. An equivalent positive and negative protein regulation was observed by SILAC/mass spectrometry, although an important up-regulation was expected after BAP1 re-expression given its deubiquinating activity. This indicates that the phenotypes observed are most likely indirect consequences of BAP1 expression modulation and catalytic function.

The pathway represented by most abundant and differentially expressed proteins was cytoskeleton organization, which was experimentally confirmed both in NCI-H226 and QR. Cell lines expressing functional BAP1 showed acquisition of stress fibers – an important decrease of N-cadherin expression concomitantly with acquired motility and invasive capacities. The N-Cadherin decrease in BAP1^wt^ cells can be puzzling, as most epithelial cancers undergo epithelial-to-mesenchymal transition and lose their E-Cadherin expression in favor of N-cadherin expression. However, other types of cancer cell migration exist, (for a review, [27]) sometimes accompanied with decrease of all cell-cell adhesion proteins and showing a single-cell or amoeboid migration. This might be the case for NCI-H226 and QR cells. Interestingly, our results suggest that mesothelioma cells expressing wild-type BAP1 are more aggressive, which is consistent with clinical studies associating BAP1 expression with worse prognosis in mesothelioma [28]. It was showed that morphological alterations and acquired invasive capacities were at least in part consequences of increased intra-cellular ROS level, as already described in other models [29]. Interestingly, morphologic changes were also observed after BAP1 expression modulation in uveal melanoma cell lines [30], although no loss of cellular identity was observed in our study.

A global decrease of mitochondrial protein pool accompanied by a decrease in respiratory capacities was observed in wild-type *BAP1*-expressing cells, suggesting a role of BAP1 in mitochondrial respiration, which was confirmed using the Seahorse technique. However, evaluation of mitochondrial membrane polarization showed divergent results between NCI-H226 and QR cell lines, suggesting that depolarization defects observed in the NCI-H226 cell line are not the leading cause of respiratory defects observed in both cell lines. Interestingly, mitochondrial defects have already been observed in mesothelioma [31], and targeting mitochondria has been proposed for mesothelioma treatments [32, 33]. In this study, antioxidant treatment partially restored the respiratory capacities, suggesting that high intracellular ROS level is at least in part responsible for the respiratory defect. These results support a relationship between increased intracellular ROS levels and oxidative phosphorylation defects, as seen in neurodegenerative diseases [26]. A recent study reported BAP1 as having a role in the maintenance of metabolic function through protein regulation [34]. It is worth noting that this study found an opposite effect, as BAP1 loss contributed to mitochondrial protein depletion. However, this study supports the finding that BAP1 plays an important role in mitochondrial functions. PGC-1α has been described as a poly-deubiquitinated BAP1 target and is stabilized by BAP1 expression [19]. However no expression variation was detected in mesothelial cell lines (Supplementary Figure 8), suggesting that PGC-1α is not involved in BAP1-mediated mitochondrial regulation.

Increased levels of intracellular ROS as well as hypersensitivity to extracellular ROS for wild-type *BAP1*-expressing cells is particularly relevant to mesothelioma. Asbestos, which is the major environmental cause of malignant pleural mesothelioma, was shown to be involved in increased ROS levels within the pleura. It was subsequently suggested that asbestos-induced increased ROS levels was a major cause of the tumorigenesis of mesothelioma [35], and *in vitro* anti-oxidant treatment decreased invasive capacities of mesothelioma tumors [36]. Together with our results, these data suggest an interaction between BAP1 expression, ROS sensitivity and asbestos exposure [26, 37].

The BAP1 tumor suppressor is inactivated in numerous cancer types that are outstanding in their molecular diversity and embryonic origin. Prognosis effect of BAP1 differs according to tumor types. In uveal melanoma, *BAP1* inactivation is associated with metastasis development and worse prognosis [2]. In clear cell renal cell carcinoma, BAP1 inactivation defines a specific subtype of cancer of worse prognosis [4]. Interestingly, in mesothelioma, *BAP1* inactivation is associated with less aggressive tumors and a better outcome [28]. This suggests that the BAP1 tumor suppressor functions are tumor and context specific, which renders the elucidation of BAP1's role even more challenging.

Our results support a major role of BAP1 in regulating cell morphology, cell migration and mitochondrial respiration in mesothelioma. Moreover, the major alteration of these pathways is observed at the proteomic level. Antioxidant treatment partially abrogated the phenotypes, supporting the sensing and/or management of ROS as the most direct function altered by BAP1 expression modulation, cellular morphology and respiration defects most likely being at least in part consequence of increased intracellular oxidative stress.

Identification of BAP1 targets involved in these functions would be crucial to decipher the mechanisms. It will also be very informative to determine whether these newly described BAP1 functions take place in other tumor types or are context dependent.

## MATERIALS AND METHODS

### BAP1 plasmids

The complete open reading frame of human wild-type *BAP1* was amplified by PCR after reverse transcription and cloned into the pCDH1 vector. This sequence corresponds to the 2190 coding nucleotides of NM_004656.3. A catalytic dead mutant was obtained by introducing the C91S mutation using the QuickChange site-directed mutagenesis kit (Agilent Genomics). Both open reading frames were subsequently cloned into the pBABE retroviral vector. Detailed constructs, sequences and vectors are available upon request.

### Cell culture

NCI-H226 cell line was bought from ATCC (https://www.atcc.org/Products/All/CRL-5826.aspx). QR (also known as MPM_33 [23]) was provided by D.J. Identity of the cell lines was verified by SNP-array profiling and by BAP1 status using western blotting. Derivatives were further verified by Sanger sequencing for *BAP1* sequences. Cell lines were cultivated in RPMI 1640 supplemented with 10% fetal calf serum (FCS) (Invitrogen), at 37°C in a humidified incubator with 5% CO_2_. Cells were passed in order to avoid more than 70% confluency.

### Virus production and infection

pCDH1 and pBABE vectors were used for overexpression of wild-type BAP1, for SILAC/MS and experimental experiment, respectively. Retroviruses were produced by co-transfection of pMD2G, pUMVC and retroviral vectors in 293T cell line. 48h and 72h post-transfection, viral supernatants were collected, filtered with a 45μm syringe filter, mixed with Polybrene (final concentration, 8μg/ml). Infection was performed by incubating the targeted cells with viral supernatants 3h at 37°C. Cells were re-selected every four passages.

### N-Acetyl-cysteine (NAC) treatment

Cells were treated with 500nM of freshly dissolved and buffered NAC (Sigma-Aldrich) during 10 days, every 2 days.

### Cell culture in SILAC media

SILAC RPMI (Pierce Biotechnology) was supplemented with 10% dialyzed fetal bovine serum (Thermo Scientific), 1% streptomycin/penicillin. The heavy media was supplemented with ^13^C6 ^15^N4-L-arginine and ^13^C6, ^15^N2-L-lysine (R_10_, K_8_). The light media was supplemented with normal L-arginine and L-lysine (R_0_, K_0_). For SILAC experiments, NCI-H226 isogenic cell lines were grown in parallel in either light or heavy media for 6 cell doublings to insure complete amino acids incorporation.

### Mass spectrometry preparation

Protein extracts were separated on SDS–PAGE gels (10%, Invitrogen) and stained with colloidal blue staining (LabSafe GEL Blue^TM^ GBiosciences). Gel slices were excised (7 bands) and proteins were reduced with 10 mM DTT prior to alkylation with 55 mM iodoacetamide. After washing and shrinking the gel pieces with 100% MeCN, a gel digestion was performed using trypsin (Promega) overnight in 25 mM NH_4_HCO_3_ at 30°C.

Peptides were extracted and analyzed by nano-LC-MS/MS using an Ultimate 3000 system (Dionex S.A.) coupled to an Orbitrap Fusion mass spectrometer (Q-OT-qIT, Thermo Fisher Scientific). Samples were loaded on a C18 precolumn (300 μm inner diameter x 5 mm; Dionex) at 20 μl/min in 5% MeCN, 0.1% TFA. After a desalting for 3 min, the precolumn was switched on the C18 column (75 μm i.d. x 50 cm, packed with C18 PepMap™, 3 μm, 100 Å LC Packings) equilibrated in solvent A (2% MeCN, 0.1% HCO_2_H). Bound peptides were eluted using a 157 min linear gradient (from 0 to 30% (v/v)) of solvent B (80% MeCN, 0.085% HCO_2_H) at a 150 nl/min flow rate and an oven temperature of 40°C. We acquired Survey MS scans in the Orbitrap on the 400-1500 m/z range with the resolution set to a value of 240,000 and a 4 × 10^5^ ion count target. Each scan was recalibrated in real time by co-injecting an internal standard from ambient air into the C-trap. Tandem MS was performed by isolation at 1.6 Th with the quadrupole, HCD fragmentation with normalized collision energy of 35, and rapid scan MS analysis in the ion trap. The MS^2^ ion count target was set to 10^4^ and the max injection time was 100 ms and only those precursors with charge state 2–7 were sampled for MS^2^. The dynamic exclusion duration was set to 60 s with a 10 ppm tolerance around the selected precursor and its isotopes. The instrument was run in top speed mode with 3 s cycles.

### Mass spectrometry analysis

Data were acquired using the Xcalibur software (v 3.0.63) and the resulting spectra were interrogated by the Mascot^TM^ Software through Proteome Discoverer (v 1.4.0.288, Thermo Scientific) with the SwissProt Homo Sapiens database (20131211, 20279 sequences). We set carbamidomethyl cysteine, oxidation of methionine, N-terminal acetylation, heavy ^13^C_6_^15^N_2_-Lysine (Lys8) and ^13^C_6_^15^N_4_-Arginine (Arg10) as variable modifications. We set specificity of trypsin digestion and allowed 2 missed cleavage sites and we set the mass tolerances in MS and MS/MS to 2 ppm and 0.5 Da, respectively. The resulting Mascot files were further processed by using myProMS (v 3.0, [38]) and the estimated false discovery rate (FDR) by automatically filtering the Mascot score of all peptide identifications was set to 1 % (DT count). For SILAC-based protein quantification, peptides XICs (Extracted Ion Chromatograms) were retrieved from Proteome Discoverer^TM^. Scale normalization was applied to compensate for mixing errors of the different SILAC cultures as described by Yang *et al*. [39]. Protein ratios were computed as the geometrical mean of related peptides. To estimate ratio significance, a t test was performed with a Benjamini–Hochberg FDR control threshold set to 0.05. All quantified proteins have at least 3 peptides quantified (all peptides selected).

### Gene expression profiling

Total RNA of SILAC-cultured isogenic NCI-H226 cell lines were extracted with NucleoSpin RNA kit (Macherey-Nagel). Total RNA were then hybridized on HG-U133 Plus 2.0 array (Affymetrix) according to the manufacturer's recommendations (http://www.expressionanalysis.com). Fluorescences were detected with GeneChip® Scanner 3000 software and expression data were extracted with GeneChip Command Console Software (AGCC) v2.0 software (Affymetrix). After brainarray normalization [40], exclusion of non-annotated probes and log_2_(n+1) expression transformation, 2139 genes with standard deviation over 0.3 and mean value over 6.5 were considered. ToppGene (toppgene.cchmc.org) was used to estimate enrichment.

### Hierarchical clustering of NCI-H226 cell line

Public data of gene expression from Cancer Cell Line Encyclopedia (https://portals.broadinstitute.org/ccle/data/browseData?conversationPropagation=begin) were used to classify NCI-H226 cell line with 1035 other cancer cell lines. Hierarchical clustering and plot were done using ‘hclust‘ function (method = “ward.D2”) from R (v3.3.2) and expression of the 500 probes with the highest variance.

### Ingenuity pathways analysis

Data sets containing Proteins and Genes determined to be differentially regulated as described in the preceding sections and corresponding expression values were then uploaded into the Ingenuity Systems application. Fisher's exact test was used to calculate a p-value indicating the probability that a particular biological function and/or disease assigned to that network was due to chance alone.

### Protein extraction and quantification

For total protein extraction, cells were collected by centrifugation at 1,500 rpm for 5 minutes and washed in phosphate buffered saline (PBS). Cell pellets were lysed in radioimmunoprecipitation assay buffer (RIPA) [100mM Tris-HCl/pH7.5, 0.1M NaCl, 1mM EDTA, 1% Triton, 0.5% sodium deoxycholate, 0.1% sodium dodecyl sulfate (SDS)], supplemented with NaF, Na_3_VO_4_, phenylmethanesulfonylfluoride (PMSF) and a cocktail of protease inhibitors (cOmplete©, Roche). For cytoplasmic and nuclear protein fractions, cells were collected by centrifugation at 1,500 rpm for 5 minutes and washed in phosphate buffered saline (PBS). Cell pellets were lysed with NE-PER™ Nuclear and Cytoplasmic Extraction Reagents (#78833) according to the manufacturer's instruction. 50μg of protein extracts were resolved by 4-15% SDS-PAGE (SDS-PAGE Mini- PROTEAN® TGX™ #456-1086 Biorad) and transferred to 0.2 μm nitrocellulose membranes (Biorad). After blocking with blocking buffer (LI-COR, Lincoln, NE, USA), mixed with 0.1% Tween-20, the membrane was incubated with primary antibodies overnight at 4°C (BAP1, Santa Cruz sc-4 sc-28383, 1/500; CDH2, Cell signaling #14215, 1/200; β-actin, Sigma-Aldrich A-5316, 1/10000, PGC1-α, Santa Cruz sc-13067, 1/500, BAF47, BD Pharmingen #612111, 1/1000, OXR1, Novus Biologicals NBP1-86393, 1/1000). Following incubation with appropriate fluorescent secondary antibody (1/10 000 mixed with 0.1% Tween-20), the immunoreactive bands were visualized using LI-COR-Odyssey infra-red scanner (LI-COR).

### Quantitative real-time PCR

Total RNA was isolated using NucleoSpin RNA Kit (Macherey-Nagel) according to the manufacturer's instructions. First-strand cDNA was synthesized from total RNA using MultiScribe^TM^ Reverse Transcriptase (Life Technology). Quantitative real-time PCR was performed by LightCycler-based SYBR Green I dye detection with UltraSYBR Mixture (CWBiotech). *Catalase (*Forward primer: 5′-ACTGGGATCTCGTTGGAAATA AC-3′ ; Reverse primer : 5′-CCTTCAGATGTGTCTGAG GATTT-3′) is studied and *TATA-box binding protein* gene (*TBP*) is used like endogenous gene (Forward primer: 5′-CTGGCCCATAGTGATCTTT-3′; Reverse primer : 5′-GCTGGAACTCGTCTCACTATTC-3′). *E*xpression was quantified by the ^2-ΔΔ^CT method.

### Immunofluorescence staining and 3D microscopy

Cell lines were plated at 5.10^4^ cells/cm^2^ in Lab-Tek 4 chamber slides (Fisher Scientific). After 24 hours, cells were washed three times with 1X TBS (tris buffered saline), fixed with 4% paraformaldehyde for 10 minutes, permeabilized with fresh TST: 0.2 % Triton X-100 / 0.2% SVF for 5 min, washed in TBS, blocked in 10% BSA for 30 min, washed briefly in TBS and then stained with phalloidin-TRITC (1:50, Sigma, P1951), BAP1 (1:100, Santa Cruz, Sc-4), CDH2 (1:200), antibodies diluted in 1X TBS 1% BSA for 2 hour. Secondary FITC-conjugated goat anti-mouse (1:400, Dako, F0479) and anti-rabbit IgG (1:100, Dako, F0205) were diluted in 1X TBS 1% BSA and applied during 45 min after one TBS washes. Slides were mounted after once wash in VectaShield (H1200, Vector Laboratories) containing DAPI for the staining of nuclei. Images were captured by using upright widefield Apotome microscope (Zeiss) equipped with a Coolsnap HQ2 camera through x63 or x40 NA 1.4 oil-immersion objective lens and driven by Axiovision software (Zeiss). Images were then analyzed and merged using ImageJ software.

For CDH2 immunofluorescence, exposure time was not fixed for NCI-H226 cells: EV: 293.6ms, N2:363.2ms and N3: 354.4ms.

### Invasion boyden chamber assay

Cellular invasiveness was studied by using Matrigel-coated Transwell-system (BD Biosciences). In all, 1.10^5^ cells were seeded in the Transwell chamber with RPMI with 0.5% FBS and the lower chamber with 10% FBS. After 24 hours, the invasive cells were stained with crystal violet (Sigma, HT90132) and counted. Data correspond to the number of invading cells calculated with 5 areas (up, down, left, right, and middle) on the bottom part of membrane from three biological replicates. A control well (without scratching) was systematically done to verify the amount of coated cells.

### Wound-healing assay and video-microscopy

Cellular migration was studied by wound healing assay. Cells were seeded 24h before the experiment in 6 well plates in order to be confluent the day of the experiment. Before the video taking, three wounds were made in each well with a sterile tip. Cells were rinsed two times with sterile PBS and then fresh RPMI. Pictures were acquired with the Leica DMIR2 microscope and the Coolsnap HQ2 (Roper Scientific) camera. Temperature was maintained at 37°C and the C0_2_ level was monitored to 5% (Life Imaging Services) under the control of MetaMorph software (Universal Imaging). Pictures were acquired every 30min during 48.5h. Pictures were analyzed using both MetaMorph and ImageJ software.

### Colony formation assay after H_2_O_2_ treatment

Cells were seeded at 2.10^5^ and treated for 8 hours with 200μM or 400μM of H_2_O_2_the day after. Cells were than trypsinized and counted, and then seeded at 7500 cells in a 10cm^2^ plates. Well seeding was checked the day after, and culture media was changed 7 days post-seeding. 14 days after seeding, cells were stained with crystal violet. Data corresponds to the number of colonies calculated with two quarters of areas and then doubled for an average of colony numbers per plate. Survival after H_2_O_2_ was calculated by doing the ratio between number of colony treated with H_2_O_2_ and non- treated number of colonies.

### Flow cytometry

CM-H_2_DCFDA probe was used for ROS measure-ment (Life technologies). 2.10^5^ cells were seeded 24h before the experiment in 6 well plates in order to reach 60-70% confluent the day of the experiment. Cells were then incubated with CM-H_2_DCFDA (10μM) for 10 minutes, respectively. After, cells were washed in PBS then trypsinized for 5min. Cells were then resuspended with PBS and 10% fetal calf serum to inactivate the trypsin. Cells were analyzed by FACS CantoTMII (BD Biosciences) through an excitation at 494 nm and emission at 520 nm. 10,000 to 20,000 events were recorded, and the mean of fluorescence intensity (MIF) was calculated on a selected healthy population. Cells were incubated with RPMI+DMSO as a control of autofluorescence and then subtracted to the MIF10000 to 20000 events were recorded, and the mean of fluorescence intensity (MIF) was calculated on a selected healthy population. Cells were incubated with RPMI+DMSO as a control of autofluorescence and then subtracted to the MIF.

For Mitochondrial Mass measurement, 2.10^5^ cells were seeded 24h before the experiment in 6 well plates in order reach to 60-70% of confluence the day of the experiment. Cells were washed twice with warmed PBS and then incubated with RPMI + 250nM MitoTracker Red CMXRos dissolved in DMSO (M-7512 Invitrogen) for 30min out of the light and at 37°C. Cells were washed twice in PBS then trypsinized for 5min. Cells were then resuspended with PBS and 10% fetal calf serum to inactivate trypsin. Cells were analyzed by FACS CantoTMII (BD Biosciences) through an excitation at 579 nm and emission at 599 nm.

The results were analyzed with FlowJo Software (v. 8.8.2).

### Spare respiratory capacities – seahorse XF-96 metabolic flux analysis

Cells were cultured on Seahorse XF-96 plates at a density of 50-70,000 cells per well. Adjustments were done in order to have comparable basal respiration. On the day of metabolic flux analysis, cells were changed to unbuffered DMEM supplemented with 10 mM glucose, incubated at 37°C in a non-CO_2_ incubator for 1 h. All medium and injection reagents were adjusted to pH 7.4 on the day of assay. Three baseline measurements of OCR were taken before and after sequential injection of reagents. The mitochondrial inhibitors used were oligomycin (1μM or 1.5μM for NCI-H226 and QR cell lines respectively), FCCP (0.5 μM or 1μM for NCI-H226 and QR cell lines respectively), and antinomycin A/rotenone (1 μM each). OCR rates were automatically calculated and recorded by the Seahorse XF-96 software. Basal respirations were not taken into account and were rather used for normalization. Spare respiratory capacities were calculated by subtracting maximal respiration (measurements 7, 8 and 9) to the basal respiration (measurements 1, 2 and 3). The percentage spare respiratory capacities are normalized with EV expressing cells.

### Glycolytic capacity, glycolytic reserve - seahorse XF-96 glycolysis analysis

Cells were cultured on Seahorse XF-96 plates at a density of 50-70,000 cells per well. On the day of glycolysis flux analysis, cells were changed to unbuffered DMEM supplemented with 10 mM glutamine, incubated at 37°C in a non-CO_2_ incubator for 1 h. All medium and injection reagents were adjusted to pH 7.4 on the day of assay. Three baseline measurements of ECAR were taken before and after sequential injection of reagents representing basal glycolysis. First injection of 10 mM glucose into media surrounding cells to measure of glycolytic rate, second 30 μM oligomycin A to estimate of glycolytic capacity in cells, third 100 mM 2-DG, a glucose analog that inhibits glycolysis to estimate of non-glycolytic acidification. ECAR rates were automatically calculated and recorded by the Seahorse XF-96 software. Glycolytic capacities capacities were calculated by subtracting maximal glycolytic capacity (measurements 7, 8 and 9) to basal measurement (measurements 1, 2 and 3). Glycolytic reserve were calculated by subtracting maximal glycolytic capacity (measurements 7, 8 and 9) to the glycolysis measurement (measurements 4, 5 and 6). The percentage Glycolytic capacities are normalized with EV expressing cells.

### Statistics

Statistical significance was assessed using the two-tailed unpaired t-test.

The significance of each difference was attributed as follow:

p≤ 0.001: ***; p ≤ 0.01: **; p ≤0.05: *

All experiments were repeated at least three times, and the illustrating experiment reflects the trend of the three independent repeats.

## SUPPLEMENTARY MATERIALS FIGURES AND TABLES










