# Supplementary material for: Modulating BAP1 expression affects ROS homeostasis, cell motility and mitochondrial function
Source: Oncotarget. 2017 Aug 3;8(42):72513–27. doi: 10.18632/oncotarget.19872 (PMC5641149; doi:10.18632/oncotarget.19872)
Supplement: Supplementary file 1 [file oncotarget-08-72513-s001.pdf]

Modulating BAP1 expression affects ROS homeostasis, cell motility and mitochondrial function

SUPPLEMENTARY MATERIALS

Supplementary Table 1: Differentially expressed proteins. This table describes the 1098 proteins that were found statistically differentially expressed after SILAC/MS experiments and after applying the filters described in the manuscript.

See Supplementary File 1

Supplementary Table 2: Protein actors of the major differential pathways. This table describes the proteins that were found statistically differentially expressed in the major pathways altered after BAP1 expression modulation and identified through IPA analysis.

See Supplementary File 2

Supplementary Table 3: Differentially expressed genes. This table describes the 734 genes that were found statistically differentially expressed after BAP1 expression modulation and after applying the filters described in the manuscript.

See Supplementary File 3

Supplementary Table 4: Differentially expressed genes encoding differentially expressed proteins. This table informs on the differentially expressed genes that encode proteins that were differentially expressed after SILAC/MS (in bold and red, N=55). not diff: not detected or not significantly differentially expressed

See Supplementary File 4

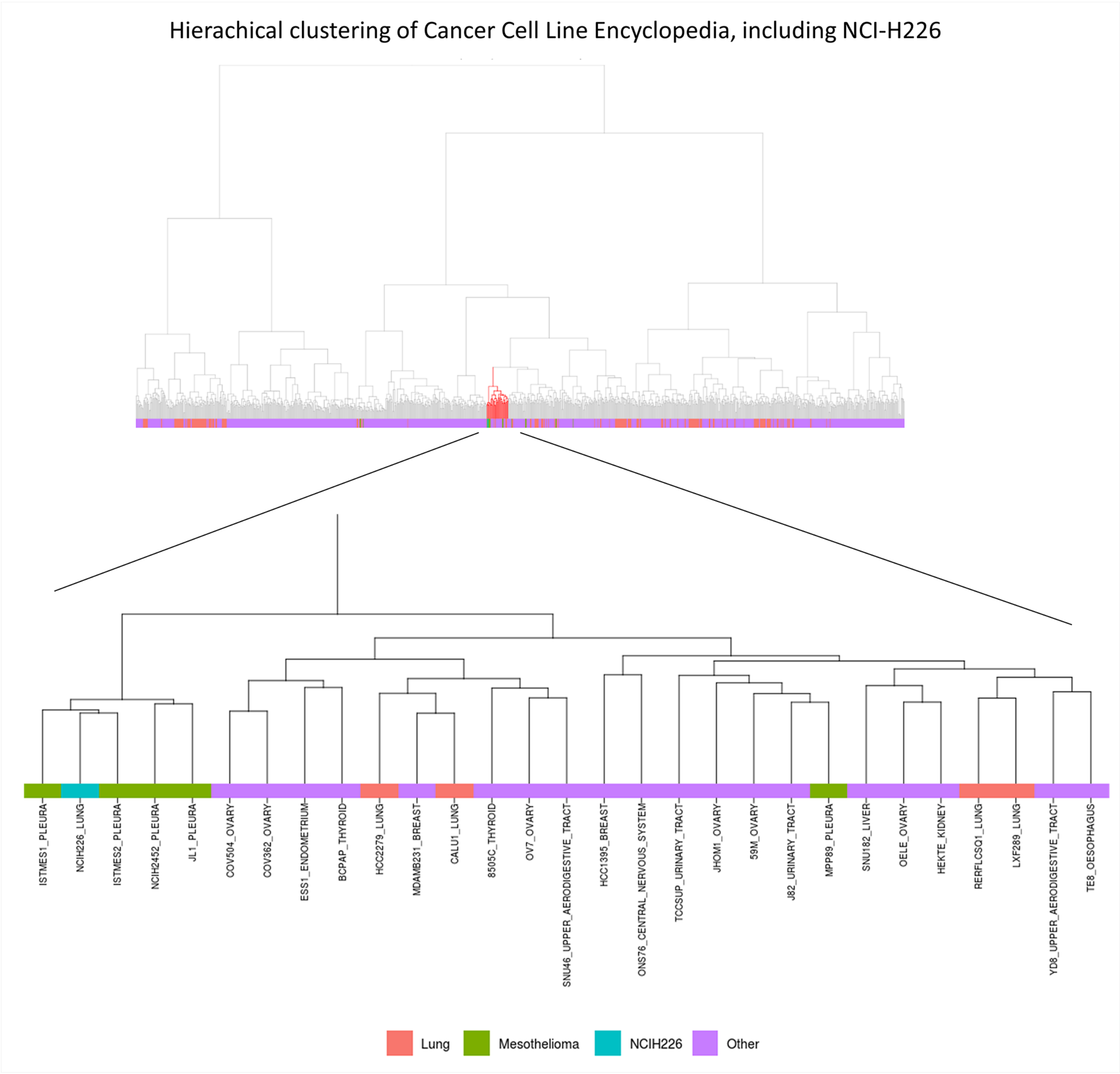

Supplementary Figure 1: Clustering of NCI-H226 cell line. Public gene expression data from the Cancer Cell Line Encyclopedia (https://portals.broadinstitute.org/ccle/home) was used to classify the NCI-H226 line among 1035 other cell lines. Hierarchical clustering and its representation have been performed using the hclust (method = "ward.D2") function of R (v3.3.2). This analysis is based of the expressions of the 500 probes which are the most differentially expressed.

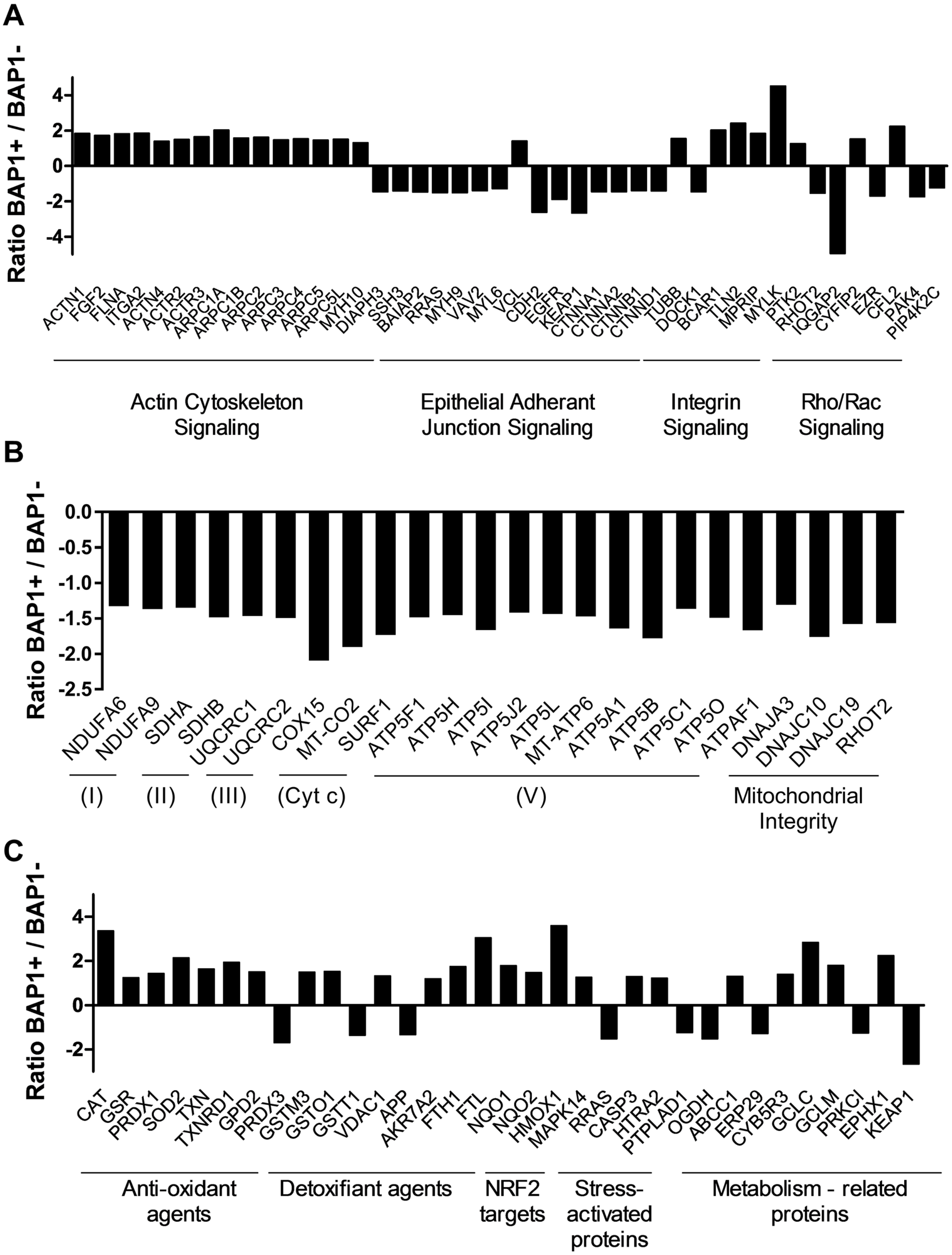

Supplementary Figure 2: Proteins involved in the major biological processes. Bar graphs representing the differentially expressed proteins involved in the major biological processes highlighted by a SILAC/MS approach. Ratio BAP1+/BAP1- reflect protein expression in a BAP1 WT expression context compared to empty vector. **(A)** Significantly differentially expressed proteins involved in actin cytoskeleton and cell motility. **(B)** Significantly differentially expressed proteins involved in mitochondrial integrity and oxidative phosphorylation. **(C)** Significantly differentially expressed proteins involved in oxidative stress level and management.

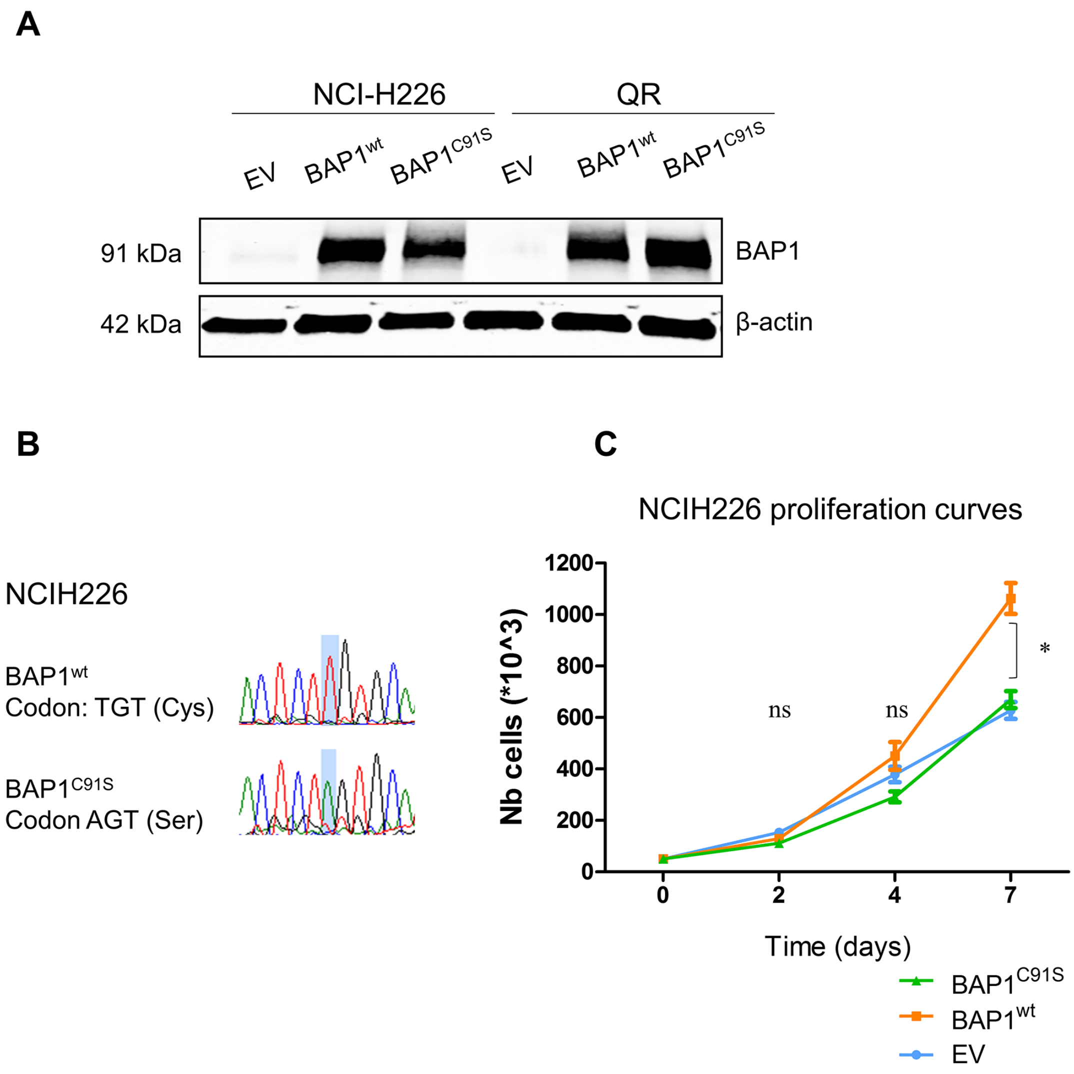

Supplementary Figure 3: Isogenic cell line assessment and properties. **(A)** Western blot quantifying BAP1 expression in NCI-H226 and QR isogenic cell lines. β-actin is used as loading control. **(B)** Sanger sequencing of BAP1 coding sequence to illustrate single nucleotide change from wild-type TGT into AGT to generate the C91S catalytically inactivating mutation in NCI-H226 cell line. **(C)** Proliferation curves of NCI-H226 isogenic cell lines performed during 7 days. Differences start to be significant at day 7 post-seeding. Data are mean ± SEM. $^*P < 0.05$, $^{**}P < 0.01$, $^{***}P < 0.001$ versus Empty Vector (EV). EV, $BAP1^{wt}$, $BAP1^{C91S}$: NCI-H226 or QR transfected with empty, wild-type BAP1 and deubiquitinase dead BAP1 vectors, respectively.

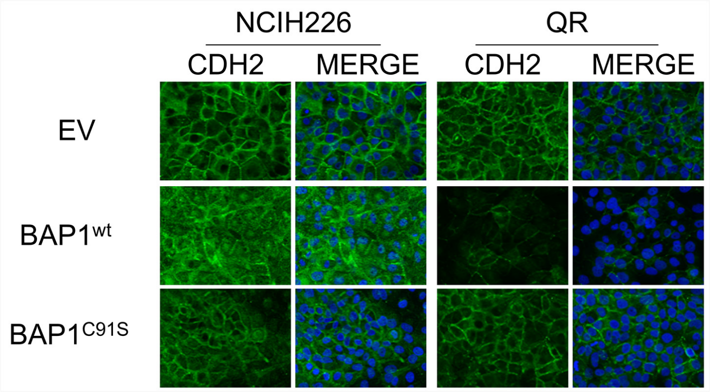

Supplementary Figure 4: Localization and organization of N-Cadherin (CDH2). Immunofluorescence of N-Cadherin (CDH2) (green). Nuclei were counterstained with DAPI (blue). Magnification x400. For NCI-H226 isogenic cell lines exposure was maximized to increase the visibility of N-Cadherin organization (Exposure time: EV: 293.6ms, N2:363.2ms and N3: 354.4ms). Consequently, intensity of signal does not reflect the protein quantity. However, for QR cell line, effort was made to keep the intensities consistent.

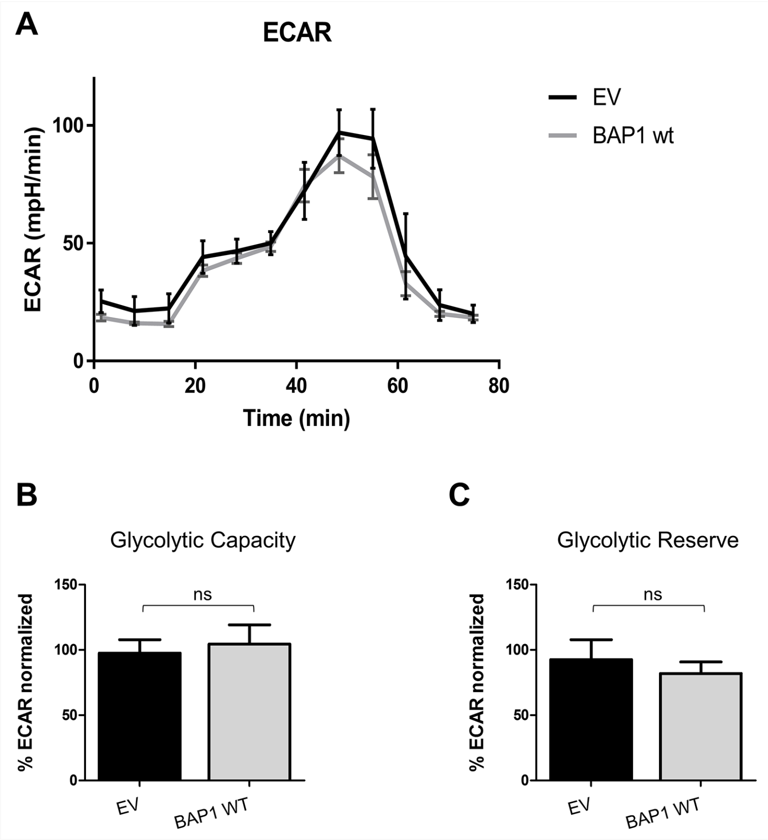

Supplementary Figure 5: Evaluation of glycolytic activity of NCI-H226 isogenic cell lines. (A) Extracellular acidification rate (ECAR), measured by the Seahorse XF-96 extracellular flux analyzer, revealing the glycolytic activity of the isogeneic cell lines. EV, BAP1^{wt}: NCI-H226 transfected with empty and wild-type BAP1, respectively. 3 reagents (10 mM glucose , 30 µM oligomycin A , 100 mM 2-DG) which are serially injected to non-glycolytic acidification, glycolysis, and glycolytic reserve, respectively.(i.e. injections). **(B** and **C)** Bar graphs are quantifications of glycolytic capacity and glycolytic reserve measurements, respectively (i.e. maximal glycolysis minus basal glycolysis and maximal glycolysis minus no glycolysis). EV, BAP1^{wt}: NCI-H226 transfected with empty, wild-type BAP1 and deubiquitinase dead BAP1 vectors, respectively.

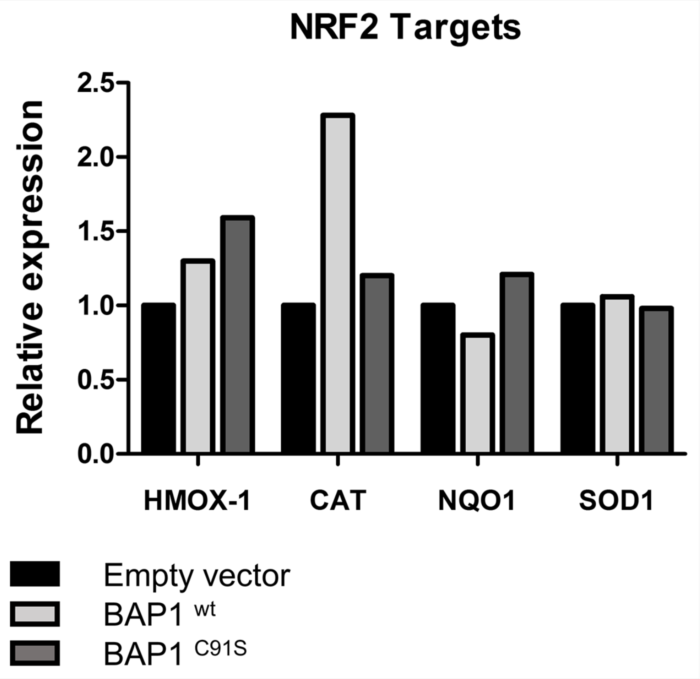

Supplementary Figure 6: NRF2 targets genes expression level. Gene expression level of *HMOX1*, *NQO1*, *CAT* and *SOD*1 measured by qPCR, normalized by *TBP* gene expression level. Normalized on non-treated EV-expressing cell line. EV, BAP1^{wt,} BAP1^{C91S}: NCI-H226 transfected with empty, wild-type BAP1 and deubiquitinase dead BAP1 vectors, respectively.

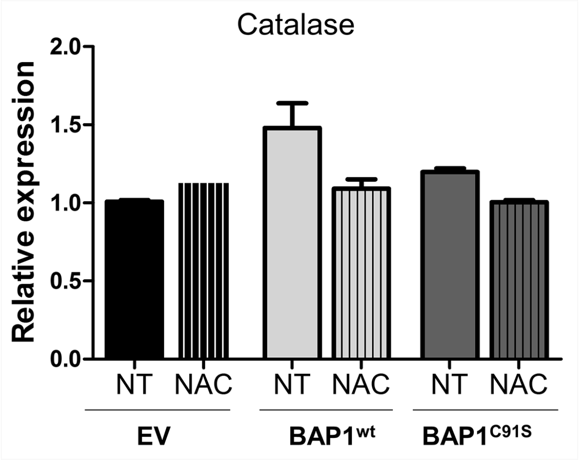

Supplementary Figure 7: Catalase expression level after N-acetyl-cysteine (NAC) treatment. *CAT* expression measured by qPCR, normalized by *TBP* gene expression level of cells treated with 500nM NAC (N-acetyl-cysteine) during 10 days versus non-treated cells (NT). Normalized on non-treated EV-expressing cell line. EV, BAP1^{wt}, BAP1^{C91S}: NCI-H226 transfected with empty, wild-type BAP1 and deubiquitinase dead BAP1 vectors, respectively.

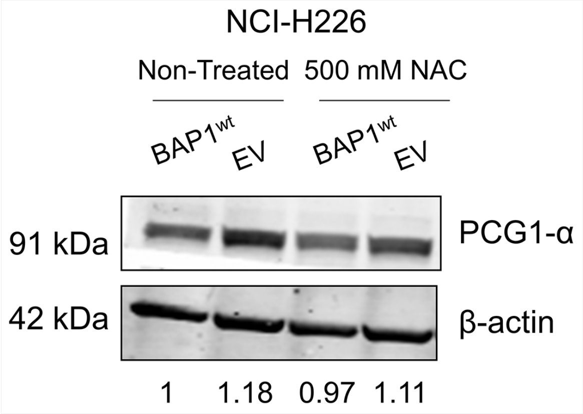

Supplementary Figure 8: Evaluation of PGC1-α protein level in NCIH226 isogenic cell line before and after NAC treatment. Western blot quantifying PGC1-α expression in NCI-H226 expressing either wild-type BAP1 or empty vector, before and after 500mM NAC treatment. β-actin is used as loading control. Quantifications are normalized on NCI-H226 expressing wild-type BAP1, without treatment.

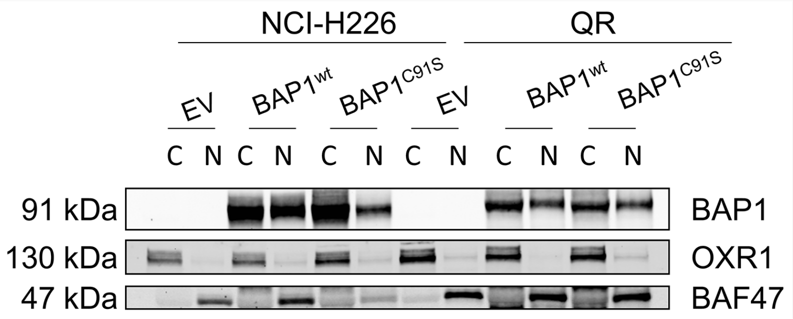

Supplementary Figure 9: BAP1 protein localization after cell fractionation. Western blot quantifying BAP1 expression in NCI-H226 and QR expressing wild-type BAP1, mutant BAP1 or empty vector after cell fractionation. C: cytoplasmic fraction, N: nuclear fraction. OXR1 is used as a control of cytoplasmic fraction, BAF47 is used as a control of nuclear fraction.
