# Supplementary material for: Modulating BAP1 expression affects ROS homeostasis, cell motility and mitochondrial function
Source: Oncotarget. 2017 Aug 3;8(42):72513–27. doi: 10.18632/oncotarget.19872 (PMC5641149; doi:10.18632/oncotarget.19872)
Supplement: Supplementary file 2 [file oncotarget-08-72513-s002.docx]

**Supplementary Table S1: Differentially expressed proteins**

This table describes the 1098 proteins that were found statistically differentially expressed after SILAC/MS experiments and after applying the filters described in the manuscript.

| **Gene Symbol** | **mean Fold Change** |
| --- | --- |
| NT5E | 17.7 |
| THBS1 | 11.9 |
| ARHGAP29 | 10.6 |
| SERPINB2 | 10.2 |
| PDGFRB | 6.5 |
| ARHGAP18 | 6.1 |
| G6PD | 6.0 |
| DBN1 | 6.0 |
| SSFA2 | 5.4 |
| TGFBI | 5.1 |
| RAPH1 | 5.1 |
| MYLK | 4.5 |
| SLC7A11 | 4.5 |
| NAV1 | 4.4 |
| ANXA6 | 4.3 |
| UBASH3B | 4.2 |
| AKR1B1 | 4.2 |
| AK4 | 4.0 |
| HMOX1 | 3.6 |
| CAT | 3.4 |
| PC | 3.3 |
| HEBP2 | 3.3 |
| FTL | 3.1 |
| TP53I3 | 3.0 |
| SERPINB1 | 3.0 |
| TMEM40 | 2.9 |
| GCLC | 2.8 |
| PGD | 2.8 |
| NAMPT | 2.8 |
| DHRS7 | 2.7 |
| TGM2 | 2.7 |
| NDRG1 | 2.7 |
| DAB2 | 2.6 |
| MYO1B | 2.6 |
| ARHGEF40 | 2.6 |
| MYO5A | 2.5 |
| ZNF185 | 2.5 |
| IMPDH2 | 2.5 |
| TLN2 | 2.4 |
| HK1 | 2.3 |
| HPS3 | 2.3 |
| CFL2 | 2.3 |
| EPHX1 | 2.2 |
| CYP24A1 | 2.2 |
| HK2 | 2.2 |
| FAM213A | 2.2 |
| CALD1 | 2.2 |
| LIMCH1 | 2.2 |
| YAP1 | 2.2 |
| PLOD2 | 2.2 |
| GJA1 | 2.2 |
| PIR | 2.2 |
| KLC2 | 2.2 |
| SLC2A1 | 2.2 |
| SOD2 | 2.1 |
| LANCL1 | 2.1 |
| C20orf27 | 2.1 |
| ASPH | 2.1 |
| YRDC | 2.1 |
| ENO2 | 2.1 |
| GDI1 | 2.0 |
| BCAR1 | 2.0 |
| SWAP70 | 2.0 |
| ARPC1A | 2.0 |
| FRMD6 | 2.0 |
| ERO1L | 2.0 |
| LASP1 | 2.0 |
| AHNAK2 | 2.0 |
| PTPN1 | 2.0 |
| NEMF | 2.0 |
| LGALS3 | 2.0 |
| SDPR | 2.0 |
| PDP1 | 2.0 |
| GNE | 2.0 |
| PPP1R18 | 2.0 |
| KCTD12 | 2.0 |
| PLCG2 | 2.0 |
| WNK1 | 2.0 |
| CKAP4 | 2.0 |
| IFI16 | 2.0 |
| SMS | 1.9 |
| TAOK3 | 1.9 |
| TXNRD1 | 1.9 |
| CORO1C | 1.9 |
| DECR1 | 1.9 |
| DYSF | 1.9 |
| YWHAZ | 1.9 |
| LAMB1 | 1.9 |
| FAM49B | 1.9 |
| EEF1D | 1.9 |
| MYO18A | 1.9 |
| CNN3 | 1.9 |
| RPIA | 1.9 |
| SLC6A6 | 1.9 |
| ADK | 1.9 |
| MIA3 | 1.9 |
| EHD2 | 1.9 |
| APPL2 | 1.9 |
| STOM | 1.9 |
| ITGA2 | 1.9 |
| THY1 | 1.9 |
| ACTN1 | 1.8 |
| MPRIP | 1.8 |
| VIM | 1.8 |
| WDHD1 | 1.8 |
| EBNA1BP2 | 1.8 |
| GNL2 | 1.8 |
| RRM2B | 1.8 |
| KIAA1429 | 1.8 |
| MYO6 | 1.8 |
| KIF3A | 1.8 |
| FAM50A | 1.8 |
| FLNA | 1.8 |
| PGM1 | 1.8 |
| ECI2 | 1.8 |
| PGK1 | 1.8 |
| GCLM | 1.8 |
| COL6A2 | 1.8 |
| NQO1 | 1.8 |
| ARL6IP5 | 1.8 |
| ATIC | 1.8 |
| 39692 | 1.8 |
| CPA4 | 1.8 |
| KIF7 | 1.8 |
| ATP6AP1 | 1.8 |
| AASS | 1.8 |
| GBE1 | 1.8 |
| SFN | 1.8 |
| FTH1 | 1.8 |
| NEDD8 | 1.8 |
| TAGLN | 1.8 |
| SORT1 | 1.7 |
| VTI1B | 1.7 |
| CAV1 | 1.7 |
| HEATR5A | 1.7 |
| RAB8B | 1.7 |
| FGF2 | 1.7 |
| SOGA1 | 1.7 |
| KTN1 | 1.7 |
| UBAP2 | 1.7 |
| LDHA | 1.7 |
| PTPN12 | 1.7 |
| CTPS1 | 1.7 |
| TMEM164 | 1.7 |
| IDH2 | 1.7 |
| MAP1B | 1.7 |
| PTGR1 | 1.7 |
| WRNIP1 | 1.7 |
| COL4A3BP | 1.7 |
| SBNO1 | 1.7 |
| LRRC20 | 1.7 |
| ACSS2 | 1.7 |
| PURA | 1.7 |
| THEM6 | 1.7 |
| ARHGEF28 | 1.7 |
| GLO1 | 1.7 |
| PDCD11 | 1.7 |
| SH3BP4 | 1.7 |
| IRAK1 | 1.7 |
| NDUFAF2 | 1.7 |
| ACTR3 | 1.7 |
| PI4K2A | 1.7 |
| HERC4 | 1.6 |
| PRKACB | 1.6 |
| KIF1C | 1.6 |
| SQRDL | 1.6 |
| ABHD14B | 1.6 |
| TXN | 1.6 |
| ARPC2 | 1.6 |
| PML | 1.6 |
| FAM3C | 1.6 |
| PSMB5 | 1.6 |
| MTHFD1 | 1.6 |
| DNAJB4 | 1.6 |
| MAP4K4 | 1.6 |
| NLN | 1.6 |
| RPRD1A | 1.6 |
| TUBA4B | 1.6 |
| SH3GLB1 | 1.6 |
| ACOT13 | 1.6 |
| FAM91A1 | 1.6 |
| STRN | 1.6 |
| NR3C1 | 1.6 |
| KDM5C | 1.6 |
| OSMR | 1.6 |
| NAP1L1 | 1.6 |
| DLGAP5 | 1.6 |
| IPO4 | 1.6 |
| TKT | 1.6 |
| ARPC1B | 1.6 |
| CAP2 | 1.6 |
| LDHAL6B | 1.6 |
| APLP2 | 1.6 |
| CTSL1 | 1.6 |
| DERL1 | 1.6 |
| CLIC1 | 1.6 |
| PLOD3 | 1.6 |
| RRAGA | 1.6 |
| TUBB | 1.6 |
| USP13 | 1.6 |
| FAM114A1 | 1.6 |
| PLCB3 | 1.6 |
| TBC1D5 | 1.6 |
| ARPC4 | 1.5 |
| HNRNPC | 1.5 |
| KPNA4 | 1.5 |
| ISYNA1 | 1.5 |
| PRDX4 | 1.5 |
| SRM | 1.5 |
| BRIX1 | 1.5 |
| RTN4 | 1.5 |
| SEC22B | 1.5 |
| CYFIP2 | 1.5 |
| GSTO1 | 1.5 |
| DYNC1LI1 | 1.5 |
| EPS15L1 | 1.5 |
| LRP1 | 1.5 |
| ALDH1B1 | 1.5 |
| ARPC5L | 1.5 |
| TRIP10 | 1.5 |
| GNPNAT1 | 1.5 |
| HPS5 | 1.5 |
| LEPREL1 | 1.5 |
| SERPINB6 | 1.5 |
| GALK1 | 1.5 |
| MTDH | 1.5 |
| ACTR2 | 1.5 |
| GPD2 | 1.5 |
| NNMT | 1.5 |
| SART1 | 1.5 |
| EIF2AK4 | 1.5 |
| GSTM3 | 1.5 |
| PCBP1 | 1.5 |
| RABGAP1L | 1.5 |
| DLAT | 1.5 |
| POP1 | 1.5 |
| CCDC88A | 1.5 |
| LGALSL | 1.5 |
| POR | 1.5 |
| STK3 | 1.5 |
| ASAP1 | 1.5 |
| IL6ST | 1.5 |
| PPFIA1 | 1.5 |
| RAB13 | 1.5 |
| ARPC3 | 1.5 |
| SORD | 1.5 |
| EMD | 1.5 |
| LRRC8D | 1.5 |
| NQO2 | 1.5 |
| PITHD1 | 1.5 |
| PPIF | 1.5 |
| PRPS2 | 1.5 |
| TMCO1 | 1.5 |
| ARPC5 | 1.5 |
| OTUD6B | 1.5 |
| SAMHD1 | 1.5 |
| PCNP | 1.5 |
| ARHGAP5 | 1.5 |
| USP11 | 1.5 |
| WNK4 | 1.5 |
| ADAM15 | 1.5 |
| KIDINS220 | 1.5 |
| MFF | 1.5 |
| RBM3 | 1.5 |
| ABCD3 | 1.5 |
| ACLY | 1.5 |
| ARL3 | 1.5 |
| PDLIM4 | 1.5 |
| MBLAC2 | 1.5 |
| PRDX6 | 1.5 |
| EHD4 | 1.4 |
| LACTB | 1.4 |
| LUZP1 | 1.4 |
| MED14 | 1.4 |
| RAI14 | 1.4 |
| BICD2 | 1.4 |
| PRDX1 | 1.4 |
| SLC4A7 | 1.4 |
| DENR | 1.4 |
| GBA | 1.4 |
| WDR77 | 1.4 |
| BZW1 | 1.4 |
| UGCG | 1.4 |
| TNPO1 | 1.4 |
| SCYL1 | 1.4 |
| ADSS | 1.4 |
| SCARB2 | 1.4 |
| ATP6V1C1 | 1.4 |
| EEF1B2 | 1.4 |
| CPOX | 1.4 |
| EIF2S1 | 1.4 |
| VCL | 1.4 |
| PRMT5 | 1.4 |
| PRDX2 | 1.4 |
| NPC1 | 1.4 |
| OLA1 | 1.4 |
| PFKP | 1.4 |
| PXDN | 1.4 |
| ACTN4 | 1.4 |
| IPO7 | 1.4 |
| PSME1 | 1.4 |
| ACAP2 | 1.4 |
| AKAP12 | 1.4 |
| AP1S1 | 1.4 |
| CYB5R3 | 1.4 |
| SLC12A4 | 1.4 |
| CCT6A | 1.4 |
| ALDH2 | 1.4 |
| DHX57 | 1.4 |
| EIF2B3 | 1.4 |
| ELMO2 | 1.4 |
| KIAA0100 | 1.4 |
| LNPEP | 1.4 |
| SLC30A1 | 1.4 |
| OSTF1 | 1.4 |
| SYPL1 | 1.4 |
| KIAA1524 | 1.4 |
| DCPS | 1.4 |
| ZMPSTE24 | 1.4 |
| MANF | 1.4 |
| SLC27A4 | 1.4 |
| PRKRA | 1.4 |
| ARF6 | 1.4 |
| DHX29 | 1.4 |
| NSUN2 | 1.4 |
| PGRMC2 | 1.4 |
| PITRM1 | 1.4 |
| RPF2 | 1.4 |
| RBM25 | 1.4 |
| CAPRIN1 | 1.4 |
| HMHA1 | 1.4 |
| NIF3L1 | 1.4 |
| CCT4 | 1.4 |
| BCAP29 | 1.4 |
| DOCK7 | 1.4 |
| NEK7 | 1.4 |
| UPF1 | 1.4 |
| KIAA1199 | 1.4 |
| PSME4 | 1.4 |
| PTDSS1 | 1.4 |
| XPNPEP1 | 1.4 |
| ARL8B | 1.4 |
| CSTB | 1.4 |
| POLA1 | 1.4 |
| ITPR3 | 1.4 |
| NTPCR | 1.4 |
| PNP | 1.4 |
| RBM39 | 1.4 |
| UBR5 | 1.4 |
| WDR1 | 1.4 |
| ZC3HAV1 | 1.4 |
| QDPR | 1.4 |
| HCFC1 | 1.4 |
| PPA1 | 1.4 |
| MYO10 | 1.4 |
| SNX9 | 1.4 |
| HEBP1 | 1.3 |
| PPP3CA | 1.3 |
| ASMTL | 1.3 |
| ATG9A | 1.3 |
| VAMP7 | 1.3 |
| EEF1G | 1.3 |
| NPEPPS | 1.3 |
| TXNDC5 | 1.3 |
| DDX54 | 1.3 |
| CYB5R1 | 1.3 |
| NUDCD1 | 1.3 |
| RBMX | 1.3 |
| ACOT7 | 1.3 |
| GFPT1 | 1.3 |
| HSD17B10 | 1.3 |
| SUPT16H | 1.3 |
| SF3A1 | 1.3 |
| SRP54 | 1.3 |
| ACAT1 | 1.3 |
| VDAC1 | 1.3 |
| XPO1 | 1.3 |
| FAM101A | 1.3 |
| SPAG9 | 1.3 |
| UCK2 | 1.3 |
| EIF2C2 | 1.3 |
| ARFGEF3 | 1.3 |
| FERMT2 | 1.3 |
| HSDL1 | 1.3 |
| TATDN1 | 1.3 |
| HSP90AB1 | 1.3 |
| PTRF | 1.3 |
| EBP | 1.3 |
| MYH10 | 1.3 |
| RSU1 | 1.3 |
| SOAT1 | 1.3 |
| PPP2R5D | 1.3 |
| HSPD1 | 1.3 |
| KCMF1 | 1.3 |
| PDLIM1 | 1.3 |
| ATP6AP2 | 1.3 |
| SLC12A9 | 1.3 |
| ZYG11B | 1.3 |
| TSTA3 | 1.3 |
| ISOC1 | 1.3 |
| PANK4 | 1.3 |
| PUS7 | 1.3 |
| UPF2 | 1.3 |
| ABCC1 | 1.3 |
| SCP2 | 1.3 |
| USP10 | 1.3 |
| AGFG1 | 1.3 |
| FLOT1 | 1.3 |
| HUWE1 | 1.3 |
| CASP3 | 1.3 |
| EIF2S3 | 1.3 |
| IFT27 | 1.3 |
| OSBPL8 | 1.3 |
| KPNB1 | 1.3 |
| YWHAQ | 1.3 |
| GNL3L | 1.3 |
| PSMB7 | 1.3 |
| PSME2 | 1.3 |
| SCRIB | 1.3 |
| SNAP29 | 1.3 |
| VPS35 | 1.3 |
| CIRH1A | 1.3 |
| DSTN | 1.3 |
| EXOC6B | 1.3 |
| FASN | 1.3 |
| RAB27A | 1.3 |
| STMN1 | 1.3 |
| PICALM | 1.3 |
| PSMA2 | 1.3 |
| SLC35E1 | 1.3 |
| SBDS | 1.3 |
| TCIRG1 | 1.3 |
| TPMT | 1.3 |
| ALDOA | 1.3 |
| ECE1 | 1.3 |
| EIF2B4 | 1.3 |
| GOLGA3 | 1.3 |
| GLT25D1 | 1.3 |
| KPNA2 | 1.3 |
| PSMA6 | 1.3 |
| HNRNPA3 | 1.3 |
| SEC24A | 1.3 |
| TRIO | 1.3 |
| VAC14 | 1.3 |
| TAF9 | 1.3 |
| MVK | 1.3 |
| PUF60 | 1.3 |
| TANC1 | 1.3 |
| UBR4 | 1.3 |
| CACYBP | 1.3 |
| LARP1 | 1.3 |
| MAPRE1 | 1.3 |
| PCK2 | 1.3 |
| SLC38A2 | 1.3 |
| STIM1 | 1.3 |
| TUBB6 | 1.3 |
| CEP170 | 1.3 |
| EPS8L2 | 1.3 |
| PTK2 | 1.3 |
| MAPK14 | 1.3 |
| NPM1 | 1.3 |
| PSMA3 | 1.3 |
| SLC12A7 | 1.3 |
| IDH1 | 1.3 |
| AP2A1 | 1.3 |
| EPS15 | 1.3 |
| PSMB3 | 1.3 |
| PSMB4 | 1.3 |
| SEC23A | 1.3 |
| ARMC6 | 1.3 |
| EXOSC10 | 1.3 |
| POFUT1 | 1.3 |
| AHNAK | 1.3 |
| ATL3 | 1.3 |
| ADAR | 1.3 |
| KIF13A | 1.3 |
| TIGAR | 1.3 |
| FKBP4 | 1.2 |
| GAPDH | 1.2 |
| GSR | 1.2 |
| QSOX1 | 1.2 |
| SLC39A14 | 1.2 |
| UBE4A | 1.2 |
| ABHD10 | 1.2 |
| ANP32E | 1.2 |
| APOO | 1.2 |
| AGTRAP | 1.2 |
| BTF3 | 1.2 |
| CLTC | 1.2 |
| MYADM | 1.2 |
| PRPF40A | 1.2 |
| PSMC6 | 1.2 |
| RPL29 | 1.2 |
| SNX12 | 1.2 |
| HECTD1 | 1.2 |
| PDCL3 | 1.2 |
| RDX | 1.2 |
| RIF1 | 1.2 |
| SF3B4 | 1.2 |
| CCDC22 | 1.2 |
| GDI2 | 1.2 |
| HINT1 | 1.2 |
| HSPA1A | 1.2 |
| KIF2C | 1.2 |
| ERBB2IP | 1.2 |
| SNX2 | 1.2 |
| TMEM2 | 1.2 |
| DUT | 1.2 |
| HPCAL1 | 1.2 |
| HTRA2 | 1.2 |
| EIF2S2 | 1.2 |
| GUK1 | 1.2 |
| RAB10 | 1.2 |
| SLK | 1.2 |
| ACAT2 | 1.2 |
| EIF2A | 1.2 |
| EMC2 | 1.2 |
| EMC3 | 1.2 |
| ESYT2 | 1.2 |
| EXOC8 | 1.2 |
| HPRT1 | 1.2 |
| RASAL2 | 1.2 |
| TALDO1 | 1.2 |
| UBA1 | 1.2 |
| CAP1 | 1.2 |
| EXOC5 | 1.2 |
| ATP13A3 | 1.2 |
| DOCK9 | 1.2 |
| PSMB2 | 1.2 |
| RNF31 | 1.2 |
| PPIP5K2 | 1.2 |
| IDH3A | 1.2 |
| IDH3G | 1.2 |
| EIF4H | 1.2 |
| PSMA1 | 1.2 |
| RRBP1 | 1.2 |
| SEC31A | 1.2 |
| TBC1D9B | 1.2 |
| AKAP9 | 1.2 |
| - | 1.2 |
| CASK | 1.2 |
| HEATR1 | 1.2 |
| HIBCH | 1.2 |
| LAMC1 | 1.2 |
| NAGK | 1.2 |
| PEBP1 | 1.2 |
| RDH11 | 1.2 |
| METAP2 | 1.2 |
| CRTAP | 1.2 |
| DDX39B | 1.2 |
| RPL4 | 1.2 |
| SCAMP3 | 1.2 |
| WWC2 | 1.2 |
| AKR7A2 | 1.2 |
| DNPEP | 1.2 |
| GBF1 | 1.2 |
| MIOS | 0.8 |
| PIP4K2C | 0.8 |
| XPOT | 0.8 |
| MMS19 | 0.8 |
| POLR3A | 0.8 |
| YKT6 | 0.8 |
| MRPL46 | 0.8 |
| STRN4 | 0.8 |
| PTPLAD1 | 0.8 |
| SNRNP40 | 0.8 |
| C12orf10 | 0.8 |
| TMED7 | 0.8 |
| TRRAP | 0.8 |
| PSAP | 0.8 |
| GLRX3 | 0.8 |
| TPM4 | 0.8 |
| TIMELESS | 0.8 |
| GAK | 0.8 |
| PRPF31 | 0.8 |
| SH3GL1 | 0.8 |
| CDC37 | 0.8 |
| CNTNAP1 | 0.8 |
| NMD3 | 0.8 |
| POLR3C | 0.8 |
| PRKCI | 0.8 |
| ATXN10 | 0.8 |
| MTX1 | 0.8 |
| ANAPC5 | 0.8 |
| LAMP1 | 0.8 |
| IL1RAP | 0.8 |
| RAB8A | 0.8 |
| VCPIP1 | 0.8 |
| SH3GLB2 | 0.8 |
| KDELR2 | 0.8 |
| PTRH2 | 0.8 |
| TMED10 | 0.8 |
| KIRREL | 0.8 |
| TWF1 | 0.8 |
| USP5 | 0.8 |
| CHID1 | 0.8 |
| EIF3B | 0.8 |
| ILF2 | 0.8 |
| ITGA5 | 0.8 |
| FAM129A | 0.8 |
| KIAA1609 | 0.8 |
| AGL | 0.8 |
| KIF14 | 0.8 |
| LMAN2 | 0.8 |
| NUP188 | 0.8 |
| RAB34 | 0.8 |
| PYCR1 | 0.8 |
| CLPTM1 | 0.8 |
| LIG4 | 0.8 |
| VASP | 0.8 |
| CMAS | 0.8 |
| YWHAH | 0.8 |
| MALT1 | 0.8 |
| PTK2B | 0.8 |
| OAT | 0.8 |
| URB2 | 0.8 |
| EIF4A2 | 0.8 |
| IRGQ | 0.8 |
| SPATA5 | 0.8 |
| MYRF | 0.8 |
| LARS2 | 0.8 |
| TEX10 | 0.8 |
| FBXL18 | 0.8 |
| MTOR | 0.8 |
| MRPL16 | 0.8 |
| ERP29 | 0.8 |
| SCAF8 | 0.8 |
| AHSA1 | 0.8 |
| UBE4B | 0.8 |
| DNAJA3 | 0.8 |
| WDR18 | 0.8 |
| RAVER1 | 0.8 |
| ITSN1 | 0.8 |
| ERAP1 | 0.8 |
| SMARCA5 | 0.8 |
| NUP210 | 0.8 |
| C16orf58 | 0.8 |
| CRYZ | 0.8 |
| LTN1 | 0.8 |
| RAP1GDS1 | 0.8 |
| SNF8 | 0.8 |
| FLNB | 0.8 |
| DIABLO | 0.8 |
| DHRS7B | 0.8 |
| SMC2 | 0.8 |
| L1CAM | 0.8 |
| MYL6 | 0.8 |
| INTS7 | 0.8 |
| RNF40 | 0.8 |
| ELP3 | 0.8 |
| ETHE1 | 0.8 |
| XPO6 | 0.8 |
| LARS | 0.8 |
| SNX5 | 0.8 |
| LSM14A | 0.8 |
| ACO1 | 0.8 |
| CDK1 | 0.8 |
| BCAT2 | 0.8 |
| ANLN | 0.8 |
| NDUFA6 | 0.8 |
| TSFM | 0.8 |
| TAB2 | 0.8 |
| CUL2 | 0.8 |
| COG7 | 0.8 |
| HNRNPD | 0.8 |
| MAN1B1 | 0.8 |
| TARS2 | 0.8 |
| TBC1D1 | 0.8 |
| IPO8 | 0.8 |
| EFNB1 | 0.8 |
| ADAM10 | 0.8 |
| HERC2 | 0.8 |
| MFI2 | 0.8 |
| URGCP | 0.8 |
| MAN1A2 | 0.7 |
| NUP88 | 0.7 |
| PPP4R2 | 0.7 |
| NOP2 | 0.7 |
| SURF4 | 0.7 |
| CHP1 | 0.7 |
| ANKRD52 | 0.7 |
| PDXDC1 | 0.7 |
| APP | 0.7 |
| EFTUD1 | 0.7 |
| CDC23 | 0.7 |
| GLG1 | 0.7 |
| INTS4 | 0.7 |
| SDHA | 0.7 |
| SGTA | 0.7 |
| CDC16 | 0.7 |
| GOLGB1 | 0.7 |
| PREPL | 0.7 |
| RDH13 | 0.7 |
| AGRN | 0.7 |
| SAE1 | 0.7 |
| INTS5 | 0.7 |
| ARAP1 | 0.7 |
| GARS | 0.7 |
| ATP5C1 | 0.7 |
| GTF3C3 | 0.7 |
| SPTLC1 | 0.7 |
| PLS3 | 0.7 |
| SMAP1 | 0.7 |
| AAAS | 0.7 |
| CDK5 | 0.7 |
| NDUFA9 | 0.7 |
| VPS25 | 0.7 |
| UTP20 | 0.7 |
| DHX37 | 0.7 |
| YARS2 | 0.7 |
| TMED5 | 0.7 |
| NUMA1 | 0.7 |
| ANXA3 | 0.7 |
| LRBA | 0.7 |
| PARN | 0.7 |
| TMED9 | 0.7 |
| YTHDF3 | 0.7 |
| PDE12 | 0.7 |
| KRT7 | 0.7 |
| AFG3L2 | 0.7 |
| GSTT1 | 0.7 |
| MCCC1 | 0.7 |
| PMPCB | 0.7 |
| SPR | 0.7 |
| POLR3B | 0.7 |
| PTK7 | 0.7 |
| MAD1L1 | 0.7 |
| PRPF19 | 0.7 |
| NT5C3 | 0.7 |
| SLC44A2 | 0.7 |
| MSI2 | 0.7 |
| DPP8 | 0.7 |
| TOR1AIP2 | 0.7 |
| IGF1R | 0.7 |
| ASNA1 | 0.7 |
| ATG2A | 0.7 |
| COG1 | 0.7 |
| IVD | 0.7 |
| CPSF3L | 0.7 |
| AACS | 0.7 |
| DDX23 | 0.7 |
| RFC3 | 0.7 |
| IMPA1 | 0.7 |
| CNN2 | 0.7 |
| TCF25 | 0.7 |
| TUBGCP2 | 0.7 |
| AARS2 | 0.7 |
| DBNL | 0.7 |
| MCMBP | 0.7 |
| WDR5 | 0.7 |
| PDS5B | 0.7 |
| MTSS1L | 0.7 |
| SLC16A1 | 0.7 |
| COG2 | 0.7 |
| TXNL1 | 0.7 |
| TELO2 | 0.7 |
| ATP5J2 | 0.7 |
| RAB12 | 0.7 |
| ALG5 | 0.7 |
| DDX47 | 0.7 |
| NMNAT1 | 0.7 |
| HSPBP1 | 0.7 |
| INTS1 | 0.7 |
| GPR126 | 0.7 |
| CTNNB1 | 0.7 |
| MVP | 0.7 |
| FAM208A | 0.7 |
| WFS1 | 0.7 |
| COG4 | 0.7 |
| ERP44 | 0.7 |
| GCA | 0.7 |
| HEATR2 | 0.7 |
| VAV2 | 0.7 |
| SCFD2 | 0.7 |
| COG8 | 0.7 |
| RPS6KA1 | 0.7 |
| SSH3 | 0.7 |
| RICTOR | 0.7 |
| SMARCA2 | 0.7 |
| SMARCA1 | 0.7 |
| NCOA7 | 0.7 |
| ATP5L | 0.7 |
| NT5C | 0.7 |
| CTNND1 | 0.7 |
| TRIP11 | 0.7 |
| EEF1A2 | 0.7 |
| VPS33B | 0.7 |
| DCXR | 0.7 |
| TMEM245 | 0.7 |
| ATP2C1 | 0.7 |
| CLIC3 | 0.7 |
| STXBP2 | 0.7 |
| ATP5H | 0.7 |
| GAS6 | 0.7 |
| GYG1 | 0.7 |
| ARFGEF1 | 0.7 |
| LXN | 0.7 |
| ANO6 | 0.7 |
| FAM83H | 0.7 |
| ABCB7 | 0.7 |
| SUN2 | 0.7 |
| CARD11 | 0.7 |
| MOV10 | 0.7 |
| MORF4L1 | 0.7 |
| UQCRC1 | 0.7 |
| MPDU1 | 0.7 |
| DLST | 0.7 |
| HSPA4L | 0.7 |
| SRPRB | 0.7 |
| BSG | 0.7 |
| MLST8 | 0.7 |
| NBAS | 0.7 |
| MT-ATP6 | 0.7 |
| TUBGCP3 | 0.7 |
| PHB | 0.7 |
| ADAM9 | 0.7 |
| COL18A1 | 0.7 |
| SDC4 | 0.7 |
| SPG20 | 0.7 |
| DCTPP1 | 0.7 |
| ATP5F1 | 0.7 |
| SDHB | 0.7 |
| CTNNA1 | 0.7 |
| DOCK1 | 0.7 |
| ATP5O | 0.7 |
| MRRF | 0.7 |
| MAN2B1 | 0.7 |
| PMPCA | 0.7 |
| CTNNA2 | 0.7 |
| UQCRC2 | 0.7 |
| DIAPH3 | 0.7 |
| UPP1 | 0.7 |
| PRKDC | 0.7 |
| TOM1L1 | 0.7 |
| VPS13A | 0.7 |
| NIP7 | 0.7 |
| BAIAP2 | 0.7 |
| RINT1 | 0.7 |
| ATP6V0A2 | 0.7 |
| BRAT1 | 0.7 |
| ABR | 0.7 |
| NUDT1 | 0.7 |
| RTN3 | 0.7 |
| DPP9 | 0.7 |
| PAWR | 0.7 |
| PARP4 | 0.7 |
| GNL1 | 0.7 |
| DCAKD | 0.7 |
| SHMT2 | 0.7 |
| RABL6 | 0.7 |
| ARHGAP17 | 0.7 |
| CENPF | 0.7 |
| GALNT7 | 0.7 |
| FANCI | 0.7 |
| ALG2 | 0.7 |
| C7orf50 | 0.7 |
| ME2 | 0.7 |
| LEMD2 | 0.7 |
| OGDH | 0.7 |
| ECI1 | 0.7 |
| SCAMP4 | 0.7 |
| RPTOR | 0.7 |
| ALG1 | 0.7 |
| RAB31 | 0.7 |
| MYH9 | 0.7 |
| ATP1B1 | 0.7 |
| STARD13 | 0.7 |
| RRAS | 0.7 |
| TMX3 | 0.7 |
| URB1 | 0.6 |
| SERPINE1 | 0.6 |
| CBX1 | 0.6 |
| SRPR | 0.6 |
| TIMM44 | 0.6 |
| FSTL1 | 0.6 |
| PSAT1 | 0.6 |
| PHB2 | 0.6 |
| ECHS1 | 0.6 |
| ACO2 | 0.6 |
| UBAC2 | 0.6 |
| APOL2 | 0.6 |
| ATP2B1 | 0.6 |
| RNF170 | 0.6 |
| ARFIP1 | 0.6 |
| RHOT2 | 0.6 |
| HDGFRP2 | 0.6 |
| ASNS | 0.6 |
| PIEZO1 | 0.6 |
| ECH1 | 0.6 |
| NIPSNAP3A | 0.6 |
| LONP1 | 0.6 |
| KRT19 | 0.6 |
| PLXNB2 | 0.6 |
| BCL2L13 | 0.6 |
| RNASEH2A | 0.6 |
| MATN2 | 0.6 |
| DNAJC19 | 0.6 |
| TMBIM6 | 0.6 |
| OXCT1 | 0.6 |
| GALNT2 | 0.6 |
| RFC1 | 0.6 |
| ROCK2 | 0.6 |
| DFFA | 0.6 |
| GTF3C4 | 0.6 |
| THBS3 | 0.6 |
| TBRG4 | 0.6 |
| NCOR2 | 0.6 |
| LMAN1 | 0.6 |
| TECR | 0.6 |
| DGKA | 0.6 |
| CDK2 | 0.6 |
| PSMD5 | 0.6 |
| FNDC3A | 0.6 |
| WDR3 | 0.6 |
| PPIA | 0.6 |
| SLC25A13 | 0.6 |
| TTC27 | 0.6 |
| ANKRD50 | 0.6 |
| CTBP2 | 0.6 |
| IREB2 | 0.6 |
| HKDC1 | 0.6 |
| COASY | 0.6 |
| DOCK10 | 0.6 |
| TOMM40 | 0.6 |
| MRI1 | 0.6 |
| VPS36 | 0.6 |
| C16orf13 | 0.6 |
| ADH5 | 0.6 |
| ALAD | 0.6 |
| FANCD2 | 0.6 |
| LAMB2 | 0.6 |
| ATP5A1 | 0.6 |
| CHMP2A | 0.6 |
| NME3 | 0.6 |
| TWF2 | 0.6 |
| TNS3 | 0.6 |
| SEZ6L2 | 0.6 |
| BCAM | 0.6 |
| ZFC3H1 | 0.6 |
| ADI1 | 0.6 |
| VAT1 | 0.6 |
| ZCCHC6 | 0.6 |
| RRM1 | 0.6 |
| TOP2A | 0.6 |
| NFKB1 | 0.6 |
| PTGIS | 0.6 |
| ATP5I | 0.6 |
| ATPAF1 | 0.6 |
| CPNE2 | 0.6 |
| ARHGEF10 | 0.6 |
| CUL7 | 0.6 |
| ANXA8L2 | 0.6 |
| ASH2L | 0.6 |
| GPR107 | 0.6 |
| PTPRF | 0.6 |
| GOLGA5 | 0.6 |
| C21orf33 | 0.6 |
| MPP6 | 0.6 |
| BYSL | 0.6 |
| SPATS2 | 0.6 |
| PTPRJ | 0.6 |
| PRDX3 | 0.6 |
| RBPMS | 0.6 |
| RASA3 | 0.6 |
| EPDR1 | 0.6 |
| HMOX2 | 0.6 |
| CNDP2 | 0.6 |
| MEST | 0.6 |
| AGFG2 | 0.6 |
| SUMF2 | 0.6 |
| SURF1 | 0.6 |
| SEC23B | 0.6 |
| EZR | 0.6 |
| PYCARD | 0.6 |
| XRCC4 | 0.6 |
| MGLL | 0.6 |
| SLC25A4 | 0.6 |
| DNAJC10 | 0.6 |
| VPS4B | 0.6 |
| PAK4 | 0.6 |
| PDLIM2 | 0.6 |
| MYEF2 | 0.6 |
| UNC93B1 | 0.6 |
| NUDT16L1 | 0.6 |
| ATP5B | 0.6 |
| PCCA | 0.6 |
| PTER | 0.6 |
| ARHGEF26 | 0.6 |
| CUL4A | 0.6 |
| THYN1 | 0.6 |
| NARS | 0.6 |
| RMDN1 | 0.6 |
| PEAK1 | 0.6 |
| GTF2F1 | 0.6 |
| PACSIN2 | 0.5 |
| CDK9 | 0.5 |
| STUB1 | 0.5 |
| EPHB2 | 0.5 |
| ILVBL | 0.5 |
| MSRA | 0.5 |
| GGA2 | 0.5 |
| USP54 | 0.5 |
| NUDCD3 | 0.5 |
| WARS | 0.5 |
| MT-CO2 | 0.5 |
| EMG1 | 0.5 |
| ZC3HC1 | 0.5 |
| OBSL1 | 0.5 |
| TBL3 | 0.5 |
| ISOC2 | 0.5 |
| EGFR | 0.5 |
| DHRS3 | 0.5 |
| ALDH5A1 | 0.5 |
| TNPO2 | 0.5 |
| PSPH | 0.5 |
| RANBP3 | 0.5 |
| OSBPL3 | 0.5 |
| ALDH1A2 | 0.5 |
| CTNNAL1 | 0.5 |
| DSE | 0.5 |
| FSCN1 | 0.5 |
| PHLDA2 | 0.5 |
| PHGDH | 0.5 |
| ELMO3 | 0.5 |
| RHPN2 | 0.5 |
| PAN2 | 0.5 |
| KIAA1468 | 0.5 |
| SNX8 | 0.5 |
| MX1 | 0.5 |
| COBLL1 | 0.5 |
| CAND2 | 0.5 |
| COX15 | 0.5 |
| LRRC16A | 0.5 |
| ZNF316 | 0.5 |
| WDR36 | 0.5 |
| EPG5 | 0.5 |
| CGN | 0.5 |
| SARM1 | 0.5 |
| FOXP1 | 0.5 |
| PAPSS1 | 0.5 |
| FAM84B | 0.4 |
| RRAD | 0.4 |
| ARAP2 | 0.4 |
| GPX1 | 0.4 |
| PPL | 0.4 |
| TAX1BP1 | 0.4 |
| ERMP1 | 0.4 |
| TTC28 | 0.4 |
| SLIT3 | 0.4 |
| PLXDC2 | 0.4 |
| TNFAIP2 | 0.4 |
| PKP2 | 0.4 |
| FAT1 | 0.4 |
| VASN | 0.4 |
| SLC7A2 | 0.4 |
| DOCK11 | 0.4 |
| DPYSL3 | 0.4 |
| SLC1A3 | 0.4 |
| LRRN4 | 0.4 |
| PRKAR1B | 0.4 |
| CDH2 | 0.4 |
| SH3TC1 | 0.4 |
| KEAP1 | 0.4 |
| NAGLU | 0.4 |
| SLC9A3R1 | 0.4 |
| PWP2 | 0.4 |
| THBS2 | 0.4 |
| DDR2 | 0.4 |
| KIAA1598 | 0.4 |
| LAMA3 | 0.3 |
| VLDLR | 0.3 |
| LRRC1 | 0.3 |
| PHLPP1 | 0.3 |
| DFNA5 | 0.3 |
| SYNE1 | 0.3 |
| F11R | 0.3 |
| DAGLB | 0.3 |
| RBM47 | 0.3 |
| MCAM | 0.3 |
| RDH10 | 0.3 |
| NNT | 0.3 |
| MMP14 | 0.3 |
| ALDH3B1 | 0.3 |
| C9orf64 | 0.3 |
| EPHA4 | 0.2 |
| MST1R | 0.2 |
| TENM3 | 0.2 |
| ANTXR1 | 0.2 |
| IQGAP2 | 0.2 |
| CNN1 | 0.2 |
| CXADR | 0.2 |
| UPK1B | 0.2 |
| CRB2 | 0.2 |
| EPPK1 | 0.2 |
| MYO1D | 0.2 |
| PROCR | 0.1 |
| KANK1 | 0.1 |
| HSD3B1 | 0.1 |
