# Supplementary material for: Modulating BAP1 expression affects ROS homeostasis, cell motility and mitochondrial function
Source: Oncotarget. 2017 Aug 3;8(42):72513–27. doi: 10.18632/oncotarget.19872 (PMC5641149; doi:10.18632/oncotarget.19872)
Supplement: Supplementary file 3 [file oncotarget-08-72513-s003.docx]

**Table S2: Protein actors of the major differential pathways**

This table describes the proteins that were found statistically differentially expressed in the major pathways altered after BAP1 expression modulation and identified through IPA analysis.

| **Gene Symbol** | **mean Protein Fold Change** |
| --- | --- |
| ITGA5 | 0.8 |
| ACTN4 | 1.4 |
| ACTR2 | 1.5 |
| ACTR3 | 1.7 |
| ARPC3 | 1.5 |
| ARPC4 | 1.5 |
| ARPC5 | 1.5 |
| ARPC1A | 2.0 |
| ARPC1B | 1.6 |
| ARPC5L | 1.5 |
| BCAR1 | 2.0 |
| EZR | 0.6 |
| MYH9 | 0.7 |
| MYL6 | 0.8 |
| PIP4K2C | 0.8 |
| RDX | 1.2 |
| RRAS | 0.7 |
| SSH3 | 0.7 |
| TLN2 | 2.4 |
| CTNNA1 | 0.7 |
| CTNNA2 | 0.7 |
| CTNNB1 | 0.7 |
| CTNND1 | 0.7 |
| TUBB | 1.6 |
| ARHGAP5 | 1.5 |
| IGF1R | 0.7 |
| PTK2B | 0.8 |
| NFKB1 | 0.6 |
| APP | 0.7 |
| ATP5B | 0.6 |
| ATP5C1 | 0.7 |
| ATP5F1 | 0.7 |
| ATP5H | 0.7 |
| ATP5J2 | 0.7 |
| ATP5L | 0.7 |
| COX15 | 0.5 |
| GSR | 1.2 |
| MT-ATP6 | 0.7 |
| MT-CO2 | 0.5 |
| SDHA | 0.7 |
| SDHB | 0.7 |
| SURF1 | 0.6 |
| UQCRC1 | 0.7 |
| VDAC1 | 1.3 |
| AKR7A2 | 1.2 |
| GCLC | 2.8 |
| GSTT1 | 0.7 |
| MAPK14 | 1.3 |
| PRDX1 | 1.4 |
| ACTN1 | 1.8 |
| ARPC2 | 1.6 |
| BAIAP2 | 0.7 |
| CFL2 | 2.3 |
| CYFIP2 | 1.5 |
| DIAPH3 | 0.7 |
| DOCK1 | 0.7 |
| FGF2 | 1.7 |
| FLNA | 1.8 |
| IQGAP2 | 0.2 |
| ITGA2 | 1.9 |
| MPRIP | 1.8 |
| MYH10 | 1.3 |
| MYLK | 4.5 |
| PAK4 | 0.6 |
| PTK2 | 1.3 |
| ROCK2 | 0.6 |
| TRIO | 1.3 |
| VAV2 | 0.7 |
| VCL | 1.4 |
| RHOT2 | 0.6 |
| VASP | 0.8 |
| CDH2 | 0.4 |
| EGFR | 0.5 |
| KEAP1 | 0.4 |
| TUBB6 | 1.3 |
| ARF6 | 1.4 |
| MAPRE1 | 1.3 |
| ASAP1 | 1.5 |
| CAV1 | 1.7 |
| PLCG2 | 2.0 |
| ANLN | 0.8 |
| KTN1 | 1.7 |
| RHPN2 | 0.5 |
| PRKCI | 0.8 |
| ATP5A1 | 0.6 |
| ATP5I | 0.6 |
| ATP5O | 0.7 |
| ATPAF1 | 0.6 |
| CASP3 | 1.3 |
| CAT | 3.4 |
| CYB5R3 | 1.4 |
| GPD2 | 1.5 |
| HSD17B10 | 1.3 |
| HTRA2 | 1.2 |
| NDUFA6 | 0.8 |
| NDUFA9 | 0.7 |
| OGDH | 0.7 |
| PRDX3 | 0.6 |
| SOD2 | 2.1 |
| UQCRC2 | 0.7 |
| ABCC1 | 1.3 |
| DNAJA3 | 0.8 |
| DNAJB4 | 1.6 |
| DNAJC10 | 0.6 |
| DNAJC19 | 0.6 |
| EPHX1 | 2.2 |
| ERP29 | 0.8 |
| FTH1 | 1.8 |
| FTL | 3.1 |
| GCLM | 1.8 |
| GSTM3 | 1.5 |
| GSTO1 | 1.5 |
| HMOX1 | 3.6 |
| NQO1 | 1.8 |
| NQO2 | 1.5 |
| PTPLAD1 | 0.8 |
| TXN | 1.6 |
| TXNRD1 | 1.9 |
