# Supplementary material for: Modulating BAP1 expression affects ROS homeostasis, cell motility and mitochondrial function
Source: Oncotarget. 2017 Aug 3;8(42):72513–27. doi: 10.18632/oncotarget.19872 (PMC5641149; doi:10.18632/oncotarget.19872)
Supplement: Supplementary file 5 [file oncotarget-08-72513-s005.docx]

**Table S4: Differentially expressed genes encoding differentially expressed proteins**

This table informs on the differentially expressed genes that encode proteins that were differentially expressed after SILAC/MS (in bold and red, N=55).

not diff: not detected or not significantly differentially expressed

| **Gene ID** | **logFC gene expression** | **Mean Protein Ratio (SILAC)** |
| --- | --- | --- |
| SNORD114-28 | -9.4 | not diff |
| SNORD114-9 | -8.7 | not diff |
| SNORD114-21 | -8.5 | not diff |
| SNORD114-1 | -8.3 | not diff |
| SNORD114-25 | -8.2 | not diff |
| SNORD114-11 | -8.1 | not diff |
| SNORD114-15 | -7.5 | not diff |
| SNORD114-26 | -7.0 | not diff |
| SNORD114-3 | -6.8 | not diff |
| SNORD114-12 | -6.4 | not diff |
| SNORD113-9 | -6.3 | not diff |
| TMEM47 | -6.0 | not diff |
| SNORD114-22 | -5.8 | not diff |
| LPHN3 | -5.2 | not diff |
| SNORD114-20 | -5.0 | not diff |
| SH3BGRL | -4.9 | not diff |
| CCND2 | -4.9 | not diff |
| SMTNL2 | -4.3 | not diff |
| ZNF100 | -3.9 | not diff |
| MYO3A | -3.9 | not diff |
| CRABP2 | -3.7 | not diff |
| CDH12 | -3.6 | not diff |
| MUC16 | -3.5 | not diff |
| SFTPD | -3.4 | not diff |
| CDH6 | -3.2 | not diff |
| SNORD113-4 | -3.1 | not diff |
| VAT1L | -3.0 | not diff |
| KDR | -3.0 | not diff |
| CYTL1 | -2.9 | not diff |
| BPIL2 | -2.9 | not diff |
| FAM70A | -2.9 | not diff |
| STON2 | -2.9 | not diff |
| GHR | -2.9 | not diff |
| KLK7 | -2.9 | not diff |
| HCN1 | -2.8 | not diff |
| LRRCC1 | -2.8 | not diff |
| LRP2 | -2.8 | not diff |
| MLLT11 | -2.8 | not diff |
| ISM2 | -2.8 | not diff |
| ANO5 | -2.8 | not diff |
| ZBED2 | -2.8 | not diff |
| RPP25 | -2.6 | not diff |
| ZNF331 | -2.6 | not diff |
| NLGN1 | -2.6 | not diff |
| PLLP | -2.6 | not diff |
| GPC4 | -2.4 | not diff |
| COL3A1 | -2.4 | not diff |
| BCAS1 | -2.4 | not diff |
| TGM1 | -2.4 | not diff |
| LOC389023 | -2.4 | not diff |
| WNT2B | -2.4 | not diff |
| GPR87 | -2.3 | not diff |
| HIST1H4D | -2.3 | not diff |
| CYP26A1 | -2.3 | not diff |
| ZNF283 | -2.3 | not diff |
| ABCG2 | -2.2 | not diff |
| ZNF468 | -2.2 | not diff |
| TRERF1 | -2.2 | not diff |
| SNORD114-17 | -2.2 | not diff |
| FGFBP1 | -2.1 | not diff |
| GJB2 | -2.1 | not diff |
| DPP10 | -2.1 | not diff |
| SYT10 | -2.1 | not diff |
| SLAIN1 | -2.1 | not diff |
| CADM1 | -2.1 | not diff |
| ST6GAL2 | -2.1 | not diff |
| CSMD3 | -2.1 | not diff |
| LRRN1 | -2.0 | not diff |
| SLC16A5 | -2.0 | not diff |
| KRT4 | -2.0 | not diff |
| SBK2 | -2.0 | not diff |
| MT1G | -2.0 | not diff |
| ELOVL2 | -2.0 | not diff |
| FLT3 | -2.0 | not diff |
| SLFN13 | -2.0 | not diff |
| BARX2 | -1.9 | not diff |
| SPANXA2 | -1.9 | not diff |
| SPANXA2 | -1.9 | not diff |
| KANK4 | -1.9 | not diff |
| SPANXB1 | -1.9 | not diff |
| SPANXB1 | -1.9 | not diff |
| **HSD3B1** | **-1.9** | **0.1** |
| DOK5 | -1.9 | not diff |
| CYP17A1 | -1.9 | not diff |
| KLHL31 | -1.8 | not diff |
| PLCB1 | -1.8 | not diff |
| EPHA6 | -1.8 | not diff |
| KCNH5 | -1.8 | not diff |
| GRB7 | -1.8 | not diff |
| MGC24103 | -1.8 | not diff |
| EFNB3 | -1.8 | not diff |
| TMEM98 | -1.8 | not diff |
| MIR554 | -1.8 | not diff |
| BNC2 | -1.8 | not diff |
| EPS8L1 | -1.8 | not diff |
| ZNF607 | -1.8 | not diff |
| **IQGAP2** | **-1.7** | **0.2** |
| NMB | -1.7 | not diff |
| SERPINB9 | -1.7 | not diff |
| ZNF738 | -1.7 | not diff |
| HIST1H2BB | -1.7 | not diff |
| C7orf46 | -1.7 | not diff |
| ZNF528 | -1.7 | not diff |
| HIST1H2BI | -1.7 | not diff |
| TRBJ2-7 | -1.7 | not diff |
| LYPD1 | -1.7 | not diff |
| CNTN5 | -1.7 | not diff |
| MGP | -1.6 | not diff |
| RBFOX1 | -1.6 | not diff |
| FAM196A | -1.6 | not diff |
| MST4 | -1.6 | not diff |
| PPP2R2B | -1.6 | not diff |
| KCNJ4 | -1.6 | not diff |
| HIST1H2BF | -1.6 | not diff |
| NRARP | -1.6 | not diff |
| RASEF | -1.6 | not diff |
| HIST1H1B | -1.6 | not diff |
| ANGPTL4 | -1.5 | not diff |
| DNM1 | -1.5 | not diff |
| GJA5 | -1.5 | not diff |
| HCN4 | -1.5 | not diff |
| **CRB2** | **-1.5** | **0.2** |
| HIST2H2AB | -1.5 | not diff |
| CA2 | -1.5 | not diff |
| MKI67 | -1.5 | not diff |
| TNFRSF8 | -1.5 | not diff |
| FAM83B | -1.4 | not diff |
| SMPDL3B | -1.4 | not diff |
| FGFR2 | -1.4 | not diff |
| GATM | -1.4 | not diff |
| PRKCG | -1.4 | not diff |
| CRIP1 | -1.4 | not diff |
| KCNMA1 | -1.4 | not diff |
| RSPO1 | -1.4 | not diff |
| ZNF618 | -1.4 | not diff |
| SCXA | -1.4 | not diff |
| HIST1H2AM | -1.4 | not diff |
| PBX1 | -1.4 | not diff |
| CDH8 | -1.3 | not diff |
| ZNF714 | -1.3 | not diff |
| NHSL1 | -1.3 | not diff |
| COBL | -1.3 | not diff |
| BAIAP2L2 | -1.3 | not diff |
| ITGB8 | -1.3 | not diff |
| TNXB | -1.3 | not diff |
| TSPY2 | -1.3 | not diff |
| HIST1H3I | -1.3 | not diff |
| GUCY1B3 | -1.3 | not diff |
| PRG2 | -1.3 | not diff |
| **PHLPP1** | **-1.3** | **0.3** |
| CCNF | -1.3 | not diff |
| SFMBT2 | -1.3 | not diff |
| **KANK1** | **-1.3** | **0.1** |
| NPR2 | -1.3 | not diff |
| TMEM151A | -1.3 | not diff |
| IGSF9 | -1.3 | not diff |
| MCTP2 | -1.3 | not diff |
| ZNF724P | -1.3 | not diff |
| CHD5 | -1.3 | not diff |
| PTPN13 | -1.3 | not diff |
| ZNF165 | -1.3 | not diff |
| KCTD8 | -1.3 | not diff |
| HSD17B7 | -1.3 | not diff |
| RBBP8 | -1.3 | not diff |
| HIST1H3F | -1.3 | not diff |
| HIST1H2AI | -1.3 | not diff |
| BRCA2 | -1.2 | not diff |
| HIST1H4L | -1.2 | not diff |
| KCNB1 | -1.2 | not diff |
| **MST1R** | **-1.2** | **0.2** |
| ZNF610 | -1.2 | not diff |
| CDCA3 | -1.2 | not diff |
| TNXA | -1.2 | not diff |
| FAM59A | -1.2 | not diff |
| ASF1B | -1.2 | not diff |
| HIST1H2BM | -1.2 | not diff |
| ZNF506 | -1.2 | not diff |
| PRRG4 | -1.2 | not diff |
| FRMD4B | -1.2 | not diff |
| DCLRE1A | -1.2 | not diff |
| C5orf34 | -1.2 | not diff |
| SPRY3 | -1.2 | not diff |
| **C9orf64** | **-1.2** | **0.3** |
| PDE6B | -1.2 | not diff |
| RAPGEF5 | -1.2 | not diff |
| NPNT | -1.2 | not diff |
| ESCO2 | -1.2 | not diff |
| HIST1H3G | -1.2 | not diff |
| **CGN** | **-1.2** | **0.5** |
| SDF2L1 | -1.2 | not diff |
| C19orf21 | -1.2 | not diff |
| RTN1 | -1.2 | not diff |
| SCNN1A | -1.2 | not diff |
| C9orf31 | -1.2 | not diff |
| E2F8 | -1.2 | not diff |
| CNIH2 | -1.2 | not diff |
| TCF7L2 | -1.2 | not diff |
| **PRKAR1B** | **-1.2** | **0.4** |
| KIF18B | -1.2 | not diff |
| KLK8 | -1.1 | not diff |
| SLC27A3 | -1.1 | not diff |
| FOXO6//FOXO6 | -1.1 | not diff |
| BCO2 | -1.1 | not diff |
| SPIN4 | -1.1 | not diff |
| SPRY3 | -1.1 | not diff |
| C16orf59 | -1.1 | not diff |
| CAMK2A | -1.1 | not diff |
| CA11 | -1.1 | not diff |
| N4BP2 | -1.1 | not diff |
| PTAFR | -1.1 | not diff |
| IGLJ4 | -1.1 | not diff |
| RASSF3 | -1.1 | not diff |
| **PROCR** | **-1.1** | **0.1** |
| CHTF18 | -1.1 | not diff |
| GLI3 | -1.1 | not diff |
| ARID3A | -1.1 | not diff |
| SNORA70F | -1.1 | not diff |
| MCTP1 | -1.1 | not diff |
| LILRB5 | -1.1 | not diff |
| RAP1GAP2 | -1.1 | not diff |
| POLQ | -1.1 | not diff |
| KDM5D | -1.1 | not diff |
| **LRRC1** | **-1.1** | **0.3** |
| **TTC28** | **-1.1** | **0.4** |
| HAUS1 | -1.1 | not diff |
| GINS4 | -1.1 | not diff |
| FBXO5 | -1.1 | not diff |
| MND1 | -1.1 | not diff |
| CARNS1 | -1.1 | not diff |
| KCNK5 | -1.1 | not diff |
| **PRKAR1B** | **-1.1** | **0.4** |
| HIST1H3B | -1.1 | not diff |
| C4orf7 | -1.1 | not diff |
| CDC45 | -1.1 | not diff |
| CCDC85C | -1.1 | not diff |
| SKA1 | -1.1 | not diff |
| **FANCD2** | **-1.1** | **0.6** |
| C9orf100 | -1.1 | not diff |
| EDN2 | -1.1 | not diff |
| MFSD2A | -1.1 | not diff |
| ZWINT | -1.1 | not diff |
| **RFC3** | **-1.1** | **0.7** |
| LOC100287175 | -1.1 | not diff |
| LOC100507307 | -1.1 | not diff |
| SNTB1 | -1.1 | not diff |
| SPC24 | -1.1 | not diff |
| ZNF383 | -1.0 | not diff |
| C9orf150 | -1.0 | not diff |
| MIR637 | -1.0 | not diff |
| ZNF85 | -1.0 | not diff |
| CHAF1A | -1.0 | not diff |
| E2F2 | -1.0 | not diff |
| **ASH2L** | **-1.0** | **0.6** |
| GPER | -1.0 | not diff |
| CENPH | -1.0 | not diff |
| CDS1 | -1.0 | not diff |
| CORO1A | -1.0 | not diff |
| CDC6 | -1.0 | not diff |
| SH3RF2 | -1.0 | not diff |
| PLCXD1 | -1.0 | not diff |
| ROGDI | -1.0 | not diff |
| C8orf84 | -1.0 | not diff |
| ENOSF1 | -1.0 | not diff |
| CYP26B1 | -1.0 | not diff |
| ADAMTS3 | -1.0 | not diff |
| SAMD10 | -1.0 | not diff |
| MBOAT1 | 0.9 | not diff |
| FGF5 | 0.9 | not diff |
| MMP9 | 0.9 | not diff |
| TCF4 | 0.9 | not diff |
| C4BPB | 0.9 | not diff |
| RGS4 | 0.9 | not diff |
| SERPINA3 | 0.9 | not diff |
| DENND2A | 1.0 | not diff |
| MIR222 | 1.0 | not diff |
| ST3GAL4 | 1.0 | not diff |
| KIAA0247 | 1.0 | not diff |
| ADAM19 | 1.0 | not diff |
| CHI3L2 | 1.0 | not diff |
| RBM24 | 1.0 | not diff |
| **KIF3A** | **1.0** | **1.8** |
| CGRRF1 | 1.0 | not diff |
| NDRG2 | 1.0 | not diff |
| NPR3 | 1.0 | not diff |
| SLC16A2 | 1.0 | not diff |
| FMNL3 | 1.0 | not diff |
| **PTGR1** | **1.0** | **1.7** |
| ID3 | 1.0 | not diff |
| **SLC6A6** | **1.0** | **1.9** |
| NAT1 | 1.0 | not diff |
| C15orf59 | 1.0 | not diff |
| PRNP | 1.0 | not diff |
| **COL6A2** | **1.0** | **1.8** |
| PLEKHO2 | 1.0 | not diff |
| STC2 | 1.0 | not diff |
| PREX1 | 1.0 | not diff |
| BMPER | 1.0 | not diff |
| FAM174A | 1.0 | not diff |
| **PLCG2** | **1.0** | **2** |
| DDX58 | 1.0 | not diff |
| SLC26A2 | 1.0 | not diff |
| ZBTB38 | 1.0 | not diff |
| CD274 | 1.0 | not diff |
| TLR3 | 1.0 | not diff |
| BBS5 | 1.0 | not diff |
| FAM171A1 | 1.0 | not diff |
| GPR173 | 1.0 | not diff |
| NRP1 | 1.0 | not diff |
| P2RX7 | 1.0 | not diff |
| **TP53I3** | **1.0** | **3** |
| FAM46A | 1.0 | not diff |
| SLC28A3 | 1.0 | not diff |
| PINK1 | 1.0 | not diff |
| KDM5B | 1.0 | not diff |
| ADAMTS12 | 1.0 | not diff |
| CD68 | 1.0 | not diff |
| RASGEF1A | 1.0 | not diff |
| NRG1 | 1.0 | not diff |
| **PDP1** | **1.0** | **2** |
| CDC42EP3 | 1.0 | not diff |
| FBXO32 | 1.0 | not diff |
| LOC100507128 | 1.0 | not diff |
| PAM | 1.0 | not diff |
| HIST1H4H | 1.0 | not diff |
| **PCK2** | **1.0** | **1.3** |
| **PRDX4** | **1.0** | **1.5** |
| JAK3 | 1.0 | not diff |
| ENGASE | 1.0 | not diff |
| NEURL | 1.1 | not diff |
| CD59 | 1.1 | not diff |
| **GBE1** | **1.1** | **1.8** |
| C14orf182 | 1.1 | not diff |
| **AK4** | **1.1** | **4** |
| USP40 | 1.1 | not diff |
| ZFYVE1 | 1.1 | not diff |
| MAPT | 1.1 | not diff |
| BCAS4 | 1.1 | not diff |
| LY96 | 1.1 | not diff |
| CYB5A | 1.1 | not diff |
| ADAM23 | 1.1 | not diff |
| CD14 | 1.1 | not diff |
| VNN1 | 1.1 | not diff |
| MSL3L2 | 1.1 | not diff |
| FAM123B | 1.1 | not diff |
| SLC46A3 | 1.1 | not diff |
| CBLB | 1.1 | not diff |
| SLC2A3 | 1.1 | not diff |
| TRAK2 | 1.1 | not diff |
| SATB2 | 1.1 | not diff |
| LOC100506583 | 1.1 | not diff |
| FLJ39051 | 1.1 | not diff |
| MPZL3 | 1.1 | not diff |
| FAM117B | 1.1 | not diff |
| DUSP10 | 1.1 | not diff |
| PRUNE2 | 1.1 | not diff |
| ZHX2 | 1.1 | not diff |
| LOC202181 | 1.1 | not diff |
| LIPA | 1.1 | not diff |
| TSPYL4 | 1.1 | not diff |
| ROR1 | 1.1 | not diff |
| KLHL15 | 1.1 | not diff |
| FAHD2B | 1.1 | not diff |
| TMEM62 | 1.1 | not diff |
| **RAB8B** | **1.1** | **1.7** |
| PHLDA1 | 1.1 | not diff |
| PAIP2B | 1.1 | not diff |
| **LEPREL1** | **1.1** | **1.5** |
| CYP20A1 | 1.1 | not diff |
| RGMB | 1.1 | not diff |
| GPSM3 | 1.1 | not diff |
| SIX4 | 1.1 | not diff |
| PHYH | 1.1 | not diff |
| ARL4C | 1.1 | not diff |
| FBXL2 | 1.1 | not diff |
| WDR45 | 1.1 | not diff |
| SFRP1 | 1.1 | not diff |
| **TMEM40** | **1.1** | **2.9** |
| RAGE | 1.1 | not diff |
| KLHDC8B | 1.1 | not diff |
| ARL15 | 1.1 | not diff |
| INHBB | 1.1 | not diff |
| IGSF8 | 1.1 | not diff |
| PLA2G4C | 1.1 | not diff |
| RRAGD | 1.1 | not diff |
| CLDN1 | 1.1 | not diff |
| PLXNA3 | 1.1 | not diff |
| ATP8B2 | 1.1 | not diff |
| ARMC9 | 1.1 | not diff |
| CDKL5 | 1.1 | not diff |
| CCDC113 | 1.1 | not diff |
| PRTG | 1.1 | not diff |
| GPR176 | 1.1 | not diff |
| **SAMHD1** | **1.1** | **1.5** |
| ECSCR//ECSCR | 1.1 | not diff |
| ANPEP | 1.1 | not diff |
| LAYN | 1.1 | not diff |
| C11orf41 | 1.2 | not diff |
| MOSPD1 | 1.2 | not diff |
| TXNRD3 | 1.2 | not diff |
| MFSD6 | 1.2 | not diff |
| BTBD19 | 1.2 | not diff |
| SRXN1 | 1.2 | not diff |
| GLT25D2 | 1.2 | not diff |
| SYNGR1 | 1.2 | not diff |
| APH1B | 1.2 | not diff |
| TRAM2 | 1.2 | not diff |
| PIGZ | 1.2 | not diff |
| CCDC85A | 1.2 | not diff |
| C1orf74 | 1.2 | not diff |
| MXRA7 | 1.2 | not diff |
| RNF144B | 1.2 | not diff |
| C20orf194 | 1.2 | not diff |
| DUSP5 | 1.2 | not diff |
| LYSMD2 | 1.2 | not diff |
| TSKU | 1.2 | not diff |
| TNFRSF11A | 1.2 | not diff |
| INSR | 1.2 | not diff |
| CSF2 | 1.2 | not diff |
| GLIPR1 | 1.2 | not diff |
| CERCAM | 1.2 | not diff |
| PLSCR4 | 1.2 | not diff |
| TRPC4 | 1.2 | not diff |
| **MX1** | **1.2** | **0.5** |
| DDX60L | 1.2 | not diff |
| PLEK2 | 1.2 | not diff |
| DYNLT3 | 1.2 | not diff |
| **PDGFRB** | **1.2** | **6.5** |
| C20orf108 | 1.2 | not diff |
| GNG4 | 1.2 | not diff |
| PORCN | 1.2 | not diff |
| SMAGP | 1.2 | not diff |
| EIF5A2 | 1.2 | not diff |
| ITGAX | 1.2 | not diff |
| PRKAA2 | 1.2 | not diff |
| PCSK1N | 1.2 | not diff |
| AMPD3 | 1.2 | not diff |
| VEGFC | 1.2 | not diff |
| FITM2 | 1.2 | not diff |
| **GJA1** | **1.2** | **2.2** |
| PSG8 | 1.2 | not diff |
| LRFN4 | 1.2 | not diff |
| TNIK | 1.2 | not diff |
| GEM | 1.2 | not diff |
| **SLC7A11** | **1.2** | **4.5** |
| TMEM156 | 1.2 | not diff |
| IL1A | 1.2 | not diff |
| LRP12 | 1.2 | not diff |
| BMP4 | 1.2 | not diff |
| THBD | 1.2 | not diff |
| PLEKHM3 | 1.2 | not diff |
| ANG | 1.2 | not diff |
| SIAE | 1.3 | not diff |
| SLC2A12 | 1.3 | not diff |
| CRLF1 | 1.3 | not diff |
| PAQR5 | 1.3 | not diff |
| **G6PD** | **1.3** | **6** |
| ESM1 | 1.3 | not diff |
| FUCA1 | 1.3 | not diff |
| DDHD1 | 1.3 | not diff |
| SNAP25 | 1.3 | not diff |
| EVI2B | 1.3 | not diff |
| MAPRE3 | 1.3 | not diff |
| PGCP | 1.3 | not diff |
| COL1A1 | 1.3 | not diff |
| **CAT** | **1.3** | **3.4** |
| TP53INP1 | 1.3 | not diff |
| BLID | 1.3 | not diff |
| SHISA4 | 1.3 | not diff |
| LMLN | 1.3 | not diff |
| NMNAT2 | 1.3 | not diff |
| DCAF4 | 1.3 | not diff |
| RAB40B | 1.3 | not diff |
| S100A3 | 1.3 | not diff |
| PYGO1 | 1.3 | not diff |
| **UBASH3B** | **1.3** | **4.2** |
| **RAPH1** | **1.3** | **5.1** |
| ITGBL1 | 1.3 | not diff |
| BMP6 | 1.3 | not diff |
| **DYSF** | **1.3** | **1.9** |
| **ITGA2** | **1.3** | **1.9** |
| EBI3 | 1.3 | not diff |
| TPCN1 | 1.3 | not diff |
| SMOC1 | 1.3 | not diff |
| MME | 1.4 | not diff |
| PLAT | 1.4 | not diff |
| SCARNA9L | 1.4 | not diff |
| IL11 | 1.4 | not diff |
| PDE7B | 1.4 | not diff |
| CROT | 1.4 | not diff |
| MANEAL | 1.4 | not diff |
| FAM126A | 1.4 | not diff |
| CMPK2 | 1.4 | not diff |
| KCNQ5 | 1.4 | not diff |
| KIAA1324 | 1.4 | not diff |
| TUBA1A | 1.4 | not diff |
| **MYLK** | **1.4** | **4.5** |
| IFIT5 | 1.4 | not diff |
| TCP11L2 | 1.4 | not diff |
| WNT5A | 1.4 | not diff |
| IFI44 | 1.4 | not diff |
| TMEM55A | 1.4 | not diff |
| ARNT2 | 1.4 | not diff |
| RAC2 | 1.4 | not diff |
| UST | 1.4 | not diff |
| TIMP4 | 1.4 | not diff |
| FBXW10 | 1.4 | not diff |
| CSF1 | 1.4 | not diff |
| MTSS1 | 1.4 | not diff |
| FAM102B | 1.4 | not diff |
| GATA2 | 1.4 | not diff |
| EIF2C4 | 1.4 | not diff |
| MORN4 | 1.4 | not diff |
| SAMD12 | 1.5 | not diff |
| CXCL2 | 1.5 | not diff |
| **HEBP2** | **1.5** | **3.3** |
| CACNG6 | 1.5 | not diff |
| XAF1 | 1.5 | not diff |
| PRKAR2B | 1.5 | not diff |
| NGF | 1.5 | not diff |
| MR1 | 1.5 | not diff |
| OLFML3 | 1.5 | not diff |
| IL17RD | 1.5 | not diff |
| ACTBL2 | 1.5 | not diff |
| FMNL2 | 1.5 | not diff |
| ACSF2 | 1.5 | not diff |
| SNAI2 | 1.5 | not diff |
| TSPAN5 | 1.5 | not diff |
| THEM4 | 1.5 | not diff |
| QPCT | 1.5 | not diff |
| CDC14A | 1.5 | not diff |
| TM4SF19 | 1.5 | not diff |
| MIRLET7A2 | 1.5 | not diff |
| MAP3K5 | 1.5 | not diff |
| MIR568 | 1.5 | not diff |
| BTBD11 | 1.5 | not diff |
| **FRMD6** | **1.5** | **2** |
| ASAH1 | 1.5 | not diff |
| TSPAN2 | 1.5 | not diff |
| SEMA4F | 1.5 | not diff |
| SIX1 | 1.5 | not diff |
| **LGALS3** | **1.5** | **2** |
| **ARHGAP18** | **1.6** | **6.1** |
| ARHGDIB | 1.6 | not diff |
| UNC13D | 1.6 | not diff |
| SYTL3 | 1.6 | not diff |
| SCRN1 | 1.6 | not diff |
| STK17B | 1.6 | not diff |
| AMIGO2 | 1.6 | not diff |
| CHI3L1 | 1.6 | not diff |
| MMP2 | 1.6 | not diff |
| CMTM7 | 1.6 | not diff |
| **DECR1** | **1.6** | **1.9** |
| LOC100132963 | 1.6 | not diff |
| GBP1 | 1.6 | not diff |
| HTR1D | 1.6 | not diff |
| PAEP | 1.6 | not diff |
| DNER | 1.6 | not diff |
| IFI30 | 1.7 | not diff |
| **DHRS7** | **1.7** | **2.7** |
| CDA | 1.7 | not diff |
| PSG4 | 1.7 | not diff |
| TMEM92 | 1.7 | not diff |
| LGR4 | 1.7 | not diff |
| SLAMF7 | 1.7 | not diff |
| HLTF | 1.7 | not diff |
| ADRB2 | 1.7 | not diff |
| IL1B | 1.7 | not diff |
| CTPS2//CTPS2 | 1.7 | not diff |
| SUSD2 | 1.7 | not diff |
| SNAR-I | 1.7 | not diff |
| STEAP2 | 1.7 | not diff |
| MID2 | 1.7 | not diff |
| PLAGL1 | 1.7 | not diff |
| TMEM200A | 1.7 | not diff |
| PPARG | 1.7 | not diff |
| NHS | 1.7 | not diff |
| TMEM133 | 1.7 | not diff |
| PCDHB16 | 1.8 | not diff |
| SRPX2 | 1.8 | not diff |
| NCRNA00118 | 1.8 | not diff |
| C7orf69 | 1.8 | not diff |
| GM2A | 1.8 | not diff |
| F2R | 1.8 | not diff |
| ATP9A | 1.8 | not diff |
| EGR1 | 1.8 | not diff |
| CD24 | 1.8 | not diff |
| UNC13B | 1.8 | not diff |
| ARRDC4 | 1.8 | not diff |
| NSAP11 | 1.8 | not diff |
| CEACAM1 | 1.8 | not diff |
| HSD17B11 | 1.8 | not diff |
| SAMD9L | 1.8 | not diff |
| **ANXA6** | **1.8** | **4.3** |
| IFI27 | 1.8 | not diff |
| PDCD1LG2 | 1.9 | not diff |
| **ARHGAP29** | **1.9** | **10.6** |
| PLA2G16 | 1.9 | not diff |
| APOBEC3G | 1.9 | not diff |
| LMO7 | 1.9 | not diff |
| DEPDC6 | 1.9 | not diff |
| NOV | 1.9 | not diff |
| PTPRN | 1.9 | not diff |
| DKK1 | 1.9 | not diff |
| CCBE1 | 1.9 | not diff |
| C4orf49 | 1.9 | not diff |
| ALOX5 | 1.9 | not diff |
| CTSF | 1.9 | not diff |
| SCN9A | 1.9 | not diff |
| NEXN | 1.9 | not diff |
| IFI6 | 1.9 | not diff |
| ARHGAP42 | 1.9 | not diff |
| **NAV1** | **1.9** | **4.4** |
| LAMB3 | 1.9 | not diff |
| CCL5 | 1.9 | not diff |
| FZD7 | 1.9 | not diff |
| VEPH1 | 1.9 | not diff |
| IFITM1 | 1.9 | not diff |
| CHML | 2.0 | not diff |
| GGT5 | 2.0 | not diff |
| C10orf25 | 2.0 | not diff |
| SARDH | 2.0 | not diff |
| OPN3 | 2.0 | not diff |
| CNRIP1 | 2.0 | not diff |
| PRCP | 2.0 | not diff |
| RARRES3 | 2.0 | not diff |
| COLEC10 | 2.0 | not diff |
| PCDHB10 | 2.0 | not diff |
| CCL26 | 2.0 | not diff |
| CGA | 2.0 | not diff |
| PLEKHA6 | 2.0 | not diff |
| HMGA2 | 2.0 | not diff |
| GRAMD3 | 2.0 | not diff |
| ACOT1 | 2.0 | not diff |
| TXNIP | 2.0 | not diff |
| ABLIM3 | 2.0 | not diff |
| FKBP7 | 2.1 | not diff |
| ADAMTS6 | 2.1 | not diff |
| RHOBTB3 | 2.1 | not diff |
| SELM | 2.1 | not diff |
| FAM43A | 2.1 | not diff |
| SOX9 | 2.1 | not diff |
| ZNF358 | 2.1 | not diff |
| EPGN | 2.1 | not diff |
| KITLG | 2.1 | not diff |
| ARHGAP24 | 2.1 | not diff |
| NCF2 | 2.1 | not diff |
| **TGFBI** | **2.1** | **5.1** |
| LRIG1 | 2.2 | not diff |
| SERPINE2 | 2.2 | not diff |
| FAM196B | 2.2 | not diff |
| MPP1 | 2.2 | not diff |
| IL8 | 2.2 | not diff |
| PSG5 | 2.2 | not diff |
| TIE1 | 2.2 | not diff |
| SULF1 | 2.2 | not diff |
| LOC100509343 | 2.2 | not diff |
| BICC1 | 2.2 | not diff |
| TNS1 | 2.3 | not diff |
| OASL | 2.3 | not diff |
| TMOD2 | 2.3 | not diff |
| TMEM171 | 2.3 | not diff |
| SERPINA1 | 2.4 | not diff |
| CPZ | 2.4 | not diff |
| AOX1 | 2.4 | not diff |
| ITGA11 | 2.4 | not diff |
| MAP2 | 2.4 | not diff |
| PTX3 | 2.4 | not diff |
| FAR2 | 2.5 | not diff |
| LOXL3 | 2.5 | not diff |
| IGFL1 | 2.5 | not diff |
| RCAN3 | 2.6 | not diff |
| SLC4A4 | 2.6 | not diff |
| IL7R | 2.6 | not diff |
| STRA6 | 2.6 | not diff |
| RSAD2 | 2.6 | not diff |
| ZNF469 | 2.7 | not diff |
| SCPEP1 | 2.7 | not diff |
| FST | 2.7 | not diff |
| RORA | 2.7 | not diff |
| PLCB2 | 2.7 | not diff |
| OAS1 | 2.7 | not diff |
| C18orf26 | 2.7 | not diff |
| SEMA3A | 2.7 | not diff |
| IFIT2 | 2.7 | not diff |
| PTPRM | 2.7 | not diff |
| CD109 | 2.8 | not diff |
| KCNQ3 | 2.8 | not diff |
| PSG2 | 2.8 | not diff |
| LOC730755 | 2.8 | not diff |
| ABCC3 | 2.8 | not diff |
| SRGN | 2.8 | not diff |
| SH3RF3 | 2.9 | not diff |
| LOC100128054 | 2.9 | not diff |
| HSD17B2 | 2.9 | not diff |
| FOXC2 | 2.9 | not diff |
| ABI3BP | 3.0 | not diff |
| ACSL5 | 3.0 | not diff |
| PCDH1 | 3.0 | not diff |
| HLA-DRB1 | 3.0 | not diff |
| SLCO2B1 | 3.0 | not diff |
| COL8A1 | 3.1 | not diff |
| ANKRD1 | 3.1 | not diff |
| C21orf96 | 3.1 | not diff |
| IFIT1 | 3.1 | not diff |
| SPOCK1 | 3.1 | not diff |
| MLPH | 3.2 | not diff |
| IFIT3 | 3.3 | not diff |
| ADAMTS16 | 3.4 | not diff |
| **NT5E** | **3.4** | **17.7** |
| **THBS1** | **3.4** | **11.9** |
| OAS2 | 3.5 | not diff |
| GLRX | 3.5 | not diff |
| **SERPINB2** | **3.5** | **10.2** |
| TNFSF18 | 3.6 | not diff |
| IGFBP5 | 3.6 | not diff |
| F2RL2 | 3.7 | not diff |
| MX2 | 3.7 | not diff |
| AKR1B10 | 3.9 | not diff |
| IL6 | 3.9 | not diff |
| HS3ST3B1 | 3.9 | not diff |
| ENG | 4.1 | not diff |
| AKR1C3 | 4.3 | not diff |
| TGFB2 | 4.4 | not diff |
| C10orf10 | 4.4 | not diff |
| IFI44L | 4.5 | not diff |
| FN1 | 4.6 | not diff |
| MYPN | 4.6 | not diff |
| LOC541472 | 4.7 | not diff |
| CYP4F11 | 4.7 | not diff |
| VIT | 4.9 | not diff |
| LOC100507488 | 6.1 | not diff |
| FPR3 | 7.2 | not diff |
| KRT34 | 8.1 | not diff |
| BAP1 | 10.5 | not diff |
